# Cost-effectiveness of health insurance among women engaged in transactional sex and impacts on HIV transmission in Cameroon: a mathematical model

**DOI:** 10.1136/bmjgh-2024-017870

**Published:** 2025-02-18

**Authors:** Kasim Allel, Henry Cust, Iliassou Mfochive, Sandie Szawlowski, Emile Nitcheu, Eric Defo Tamgno, Stephanie Moyoum, Julienne Noo, Serge Billong, Ubald Tamoufe, Aurelia Lepine

**Affiliations:** 1 University College London, London, UK; 2 Nuffield Department of Primary Care Health Sciences, University of Oxford, Oxford, UK; 3 Duke University, Durham, North Carolina, USA; 4 John Hopkins Cameroon Program, Yaounde, Cameroon; 5 University of Yaounde, Yaoundé, Cameroon

**Keywords:** Health insurance, HIV, Mathematical modelling, Health economics

## Abstract

**Introduction:**

HIV prevalence disproportionately affects high-risk populations, particularly female sex workers in Africa. Women and girls engaging in transactional sex (WGTS) face similar health risks from unsafe practices, economic vulnerabilities and stigma. However, they are not recognised.

**Methods:**

Using existing literature and data from the POWER randomised controlled trial, we developed a deterministic compartmental model to assess HIV dynamics among WGTS, their sugar daddies and low-risk populations. We evaluated the cost-effectiveness of a new structural intervention to prevent HIV among WGTS in urban Cameroon by reducing the financial need to engage in transactional sex in the case of illness and injury shocks to the household. The intervention provided free healthcare to WGTS and their economic dependents through a zero-cost health insurance package. We explored the cost-effectiveness of this intervention considering various population coverage levels (0%, 25%, 50%, 75% and 100%). We calculated the incremental cost-effectiveness ratio (ICER) per disability-adjusted life-year (DALY) and HIV infections averted, employing both univariable and global sensitivity analyses. Probabilistic sensitivity analyses considered all parameters, including the insurance effect in reducing HIV, comparing simulated ICERs to willingness-to-pay thresholds. We also compared the health insurance strategy with expanding pre-exposure prophylaxis (PrEP) coverage. All costs were evaluated in 2023 UK pounds (£) using a 3% discount rate, with Cameroon’s gross domestic product (GDP) per capita recorded at £1239.

**Results:**

Implementing health insurance coverage levels of 25%, 50%, 75% and 100% yielded ICERs/DALY averted of £2795 (£2483—£2824), £2541 (£2370—£2592), £2263 (£2156—£2316) and £1952 (£1891—£1998), respectively, compared with 0% coverage. Probabilistic sensitivity analysis indicated an ICER=£2128/DALY averted at 100% coverage, with 58% of simulations showing ICERs<GDP per capita. Maintaining health insurance’s effect in reducing HIV above 70% could provide significant health and economic benefits. However, antiretroviral therapy (ART) efficacy significantly impacted HIV infection prevention (partial rank correlation coefficient=−0.62, p<0.001) in global sensitivity analyses; expanding ART could reduce the cost-effectiveness of health insurance. While PrEP alone is not cost-effective, combining 20% PrEP coverage with 75%–100% health insurance for WGTS maximises DALYs averted (ICER/DALY averted=£2436–£2102) and reduces infections.

**Conclusion:**

A comprehensive health insurance scheme for women in Cameroon could significantly reduce HIV infections and DALYs, promoting a more inclusive and targeted healthcare policy for women at high risk of HIV.

**WHAT IS ALREADY KNOWN ON THIS TOPIC**

**WHAT THIS STUDY ADDS**

- This study demonstrates the broad health and economic benefits of scaling up health insurance for women and girls engaging in transactional sex (WGTS) in Cameroon.
- By using a compartmental model, it examines the population-level effects, showing that health insurance coverage reduces HIV infections in the general population by preventing risky sexual behaviours among WGTS and their partners.
- These findings provide strong support for incorporating health insurance into HIV prevention strategies for WGTS, showing potential for significant public health impact.

**HOW THIS STUDY MIGHT AFFECT RESEARCH, PRACTICE OR POLICY**

- The study highlights the potential of structural interventions like health insurance in reducing HIV rates among vulnerable populations via preventing risky sexual behaviours, making these interventions more cost-effective than traditional methods alone, such as pre-exposure prophylaxis and antiretroviral therapy.
- Findings support the integration of health insurance into HIV prevention efforts to advance Sustainable Development Goals (SDGs), including SDG 3.8 (universal health coverage) and SDG 3.3 (ending the AIDS epidemic).
- This research advocates for a broader application of inclusive healthcare policies, emphasising the importance of structural approaches in achieving long-term public health goals.

## Introduction

 HIV remains a leading cause of death and disease burden in sub-Saharan Africa, disproportionately affecting women.^1^ In 2020, 63% of all new HIV infections in the region occurred in women, with 67% of these among adolescent and young women aged between 15 and 19 years.^2^ Growing evidence suggests that commercial and transactional sex are key drivers of the HIV epidemic, contributing to significant gender disparities in new HIV infections in sub-Saharan Africa.^3 4^ For instance, women engaged in commercial sex in sub-Saharan Africa are 26 times more likely to contract HIV than their peers. In Cameroon, HIV prevalence is alarmingly high, reaching 4.3% among adults and a staggering 36.5% among female sex workers (FSWs).

Commercial and transactional sex can be used as a risk-coping strategy to cope with negative economic shocks (eg, job losses, out-of-pocket health expenses and expensive religious celebrations).^5 6^ These activities enable women to establish a network of sexual partners that can provide financial support during periods of economic vulnerability. In response to such shocks, women often turn to these relationships for immediate economic relief. Within these relationships they engage in high HIV-risk behaviours, such as condomless sex, due to the risk premium associated with these actions they earn more money. This premium varies, ranging from 9% in Kenya to 66% in India.^3 4 7^ Evidence shows that negative economic shocks lead to an increase in both commercial and transactional relationships, along with riskier sexual behaviours within such relationships.^5^

Often overlooked in explaining the gender disparities are women and girls engaging in transactional sex (WGTS). Transactional sex is defined as relationships that are ‘non-commercial, non-marital sexual relationships motivated by the implicit assumption that sex will be exchanged for material support or other benefits’.^8^ WGTS typically do not identify as FSW, they have fewer sexual partners whom they refer to as ‘sugar daddies’ (or ‘papy’ in Cameroon) or sometimes as boyfriends.^9^ Transactional sex and sex work share similarities, yet are treated differently both socially and legally in Cameroon.^10^ Transactional sex, defined as non-commercial, non-marital sexual relationships where material support is implicitly exchanged for sex, is more socially accepted.^11^ Both transactional sex and sex work involve the exchange of sex for economic benefit, and both expose women to high HIV risk. Unlike formal sex work, it is not criminalised, and the stigma attached to transactional sex is less severe than that faced by FSWs. However, despite the similarities in risk, WGTS are not recognised as a key population in HIV prevention programmes.^12^ As a result, they are excluded from government initiatives like pre-exposure prophylaxis (PrEP) that are available to FSWs.

While HIV interventions, through the National HIV/AIDS Strategic Plan,^13^ in Cameroon have partly focused on FSWs in Yaoundé and overlooked WGTS.^14^ Structural economic interventions that reduce economic vulnerability and increase economic empowerment of women who rely on risky sex as a coping strategy have shown great promise in preventing HIV in sub-Saharan Africa.^15^,^18^ Recent research has demonstrated that health coverage for illnesses and accidents, the most common and impactful negative economic shock among women in Yaoundé, was highly effective at preventing HIV among trial participants.^19^ There are few studies of structural interventions to address HIV in this population and fewer with cost-effective estimates. Baird *et al* provide the most important showing the impact of cash transfers for adolescent and young women to prevent HIV but calculate relatively high cost-effectiveness between US$5000 and US$12 500 per HIV infection averted.^17^ Recognising the shared risks between these two groups is crucial to addressing HIV transmission more effectively. Expanding HIV prevention programmes to include WGTS could close a significant gap in efforts to reduce HIV infections among high-risk women in Cameroon.

**Figure 1:**
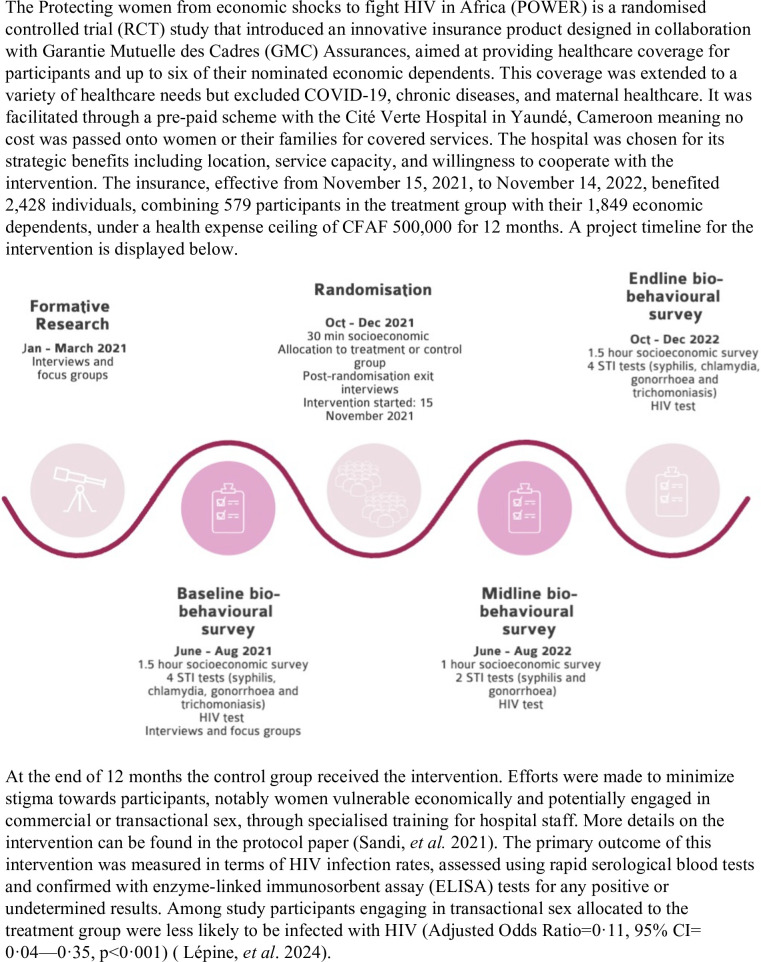
A health insurance intervention among WGTS in Yaoundé, Cameroon. Article providing the POWER results (Lépine *et al*^19^); Protocol paper (Sandie *et al*^21^). WGTS, women and girls engaging in transactional sex.

In this article, we estimated the cost-effectiveness of scaling up this intervention at the national level, using compartmental modelling to assess its impact on HIV prevention among WGTS and the general population. The study aims to demonstrate the importance of providing health insurance for optimising resource allocation in HIV spending in high-burden settings. Figure 1 provides further details on the intervention.^19^

## Methods

### Model structure

We conducted a deterministic compartmental model to analyse the impact of a health insurance scheme on HIV transmission among WGTS, their sugar daddies and their sexual partners (online supplemental figures A1–2). Targeting mechanisms and demand creation for WGTS health insurance were excluded from the model. Building on a previous model,^20^ we divided the adult heterosexual population (age 15–64 years) into four risk groups: lower-risk females (*i*=1) and males (*i*=2), sugar daddies (*i*=3) and WGTS (*i*=4). Lower-risk individuals refer to men and women who are not engaged in transactional sex. Lower-risk females enter transactional sex at a rate *k* and continue to engage in the practice for a duration of 1/γ years. Male sugar daddies transition from the lower-risk male group at rate *z* and remain as sugar daddies for a period of 1/g years before returning to the lower-risk male group. The model incorporates HIV transmission in the context of the following partnership types: main between men and women (ie, committed and often inclusive relationship; k=1), casual between men and women (k=2) and transactional sex between WGTS and sugar daddies only (k=3). The model evaluates HIV infection and considers health insurance coverage and its effect on HIV prevalence, which were allocated to WGTS and their economic dependents.^5^ We considered PrEP uptake for WGTS, disease progression, and antiretroviral therapy (ART) for any HIV-positive cases. WGTS susceptible to HIV initiate PrEP at variable rates, categorised initially by adherence level, and PrEP stops if the person contracts HIV. ART reduces HIV-related mortality and HIV infectivity. Individuals can only start ART after HIV infection. Condom usage was also considered in the model for each group. We added efficacy among PrEP, ART and condom use. The main parameter of the model was the health insurance coverage among WGTS (*s,* with ‘*s’ ∃* 0≤*s* <1) and its effectiveness in reducing HIV prevalence among WGTS ($e_{s}$), which directly impacts transmission between WGTS and the other population group.

Model specifics are detailed in online supplemental figures A1–2and text A1. Online supplemental text A2 contains our model’s differential equations.

### Model parametrisation and fitting

Our model was parametrised to epidemiological data considering the impacts of a health insurance intervention on HIV infections, based on a randomised control trial: the POWER study (Protecting women from economic shocks to fight HIV in Africa).^19 21^ The POWER study and literature^12 20 22^ provided detailed insights into sexual behaviours, condom usage trends, HIV prevalence, ART and PrEP coverage. We assumed 0% PrEP coverage in the base case scenario because in Cameroon only FSWs, injecting drug users and men who have sex with men (MSM) are eligible to initiate PrEP. We used different ‘*s’* as coverage, including 0%, 25%, 50%, 75% and 100% of WGTS populations, and the effect of the health insurance in reducing HIV was extracted from the POWER study ($e_{s}$=0.89, that is, the health insurance reduces the likelihood of acquiring HIV by 89% among treated WGTS in comparison to untreated WGTS).^19^ National studies were used to estimate the size of the population of WGTS, and sugar daddy numbers were estimated indirectly. Due to limited national-level estimates of WGTS populations, we used FSW data,^23^ incorporating variations in sensitivity analyses. Online supplemental tables A1–3 display the parameters and baseline conditions of the model. The transmission parameter was calibrated to the incidence of HIV, performed through Brent’s optimisation methods against population size and HIV data from 2001 until 2022 (online supplemental table A4).^24^ Our model evaluated the annual dynamics over a time horizon of 50 years.

### Impact analysis and cost-effectiveness

We followed the Consolidated Health Economic Evaluation Reporting Standards (CHEERS).^25^ We estimated HIV infections prevented by the intervention yearly among WGTS and in the general population (15–64 years) using a health-system perspective. Health insurance costs were estimated at £80 per person/year since this was the estimated unit price of the intervention (online supplemental table A2). The health insurance package covered free access to consultations, medications, doctor’s fees and hospitalisations for non-chronic illnesses, without copayments (figure 1). ART and PrEP are provided by the health system at no cost to the population, and our simulated health insurance schemes did not cover ART/PrEP, as it is granted by the government separately. More details are found elsewhere.^19^ We compared outcomes against a scenario without health insurance. We evaluated the cost-effectiveness of the health insurance scheme with disability-adjusted life-years (DALYs) and HIV-associated infections averted. The incremental cost-effectiveness ratio (ICER) was calculated as the ratio of discounted incremental costs to infections and HIV-related DALYs averted. DALYs and costs were extracted from the literature (online supplemental table A2). We used 2023 £ and discounted DALYs and costs at 3%. We computed 95%CIs by adjusting our models to Cameroon HIV prevalence lower and upper bounds (2.3%–3.1%).^24^

### Sensitivity and scenario analyses

We conducted a univariable sensitivity analysis to model the uncertainty effect on the number of WGTS in the population using 95% CIs (0.47% to 3.36% of the population, as there is no official data^23^), health insurance costs (£40 or £120 per person/year), the effect of the health insurance in reducing HIV (80% or 100%), discount rate (0% and 6%), ART coverage among HIV positive WGTS (35% and 79% following lower bound and general population’s coverage) and PrEP coverage among HIV negative WGTS assuming different coverage levels (20%, 40%, 60%, 80% and 100%) and combined with health insurance. We performed probability sensitivity analyses using 1000 random samples, and all parameters were varied by ±10% of their original values, including health insurance effectiveness (all proportions were capped at 0/1). We estimated cost-effectiveness by DALYs and infections averted and the percentage of cost-effective (ICER<willingness-to-pay ‘WTP’ thresholds) simulations per strategy at different WTP thresholds, considering Cameroon’s gross domestic product (GDP) per capita ≈£1,239.^26^ We also employed a global sensitivity analysis to systematically evaluate the influence of all input parameters on the number of HIV infections. We used the Latin hypercube sampling method with 1000 simulations by varying all parameters in ±10%, proportions capped at 0/1, and calculated the Spearman’s partial rank correlation coefficient (PRCC, −1<PRCC<0 indicates inverse relationship and 0<PRCC<1 a positive correlation).^27^ We only presented the parameters having a significant impact on HIV infections (hypothesis test of the correlation value with p<0.05). Finally, we conducted bivariable sensitivity analyses by modifying health insurance coverage and its effectiveness parameters simultaneously to evaluate their impacts on HIV infections.

All statistical analyses were performed in R V.3.3.4. The code is available at https://bit.ly/4bzTpWb.

### Patient and public involvement

Patients and the public were not directly involved in this study.

## Results

### Base-case cost-effectiveness results

**Figure 2:**
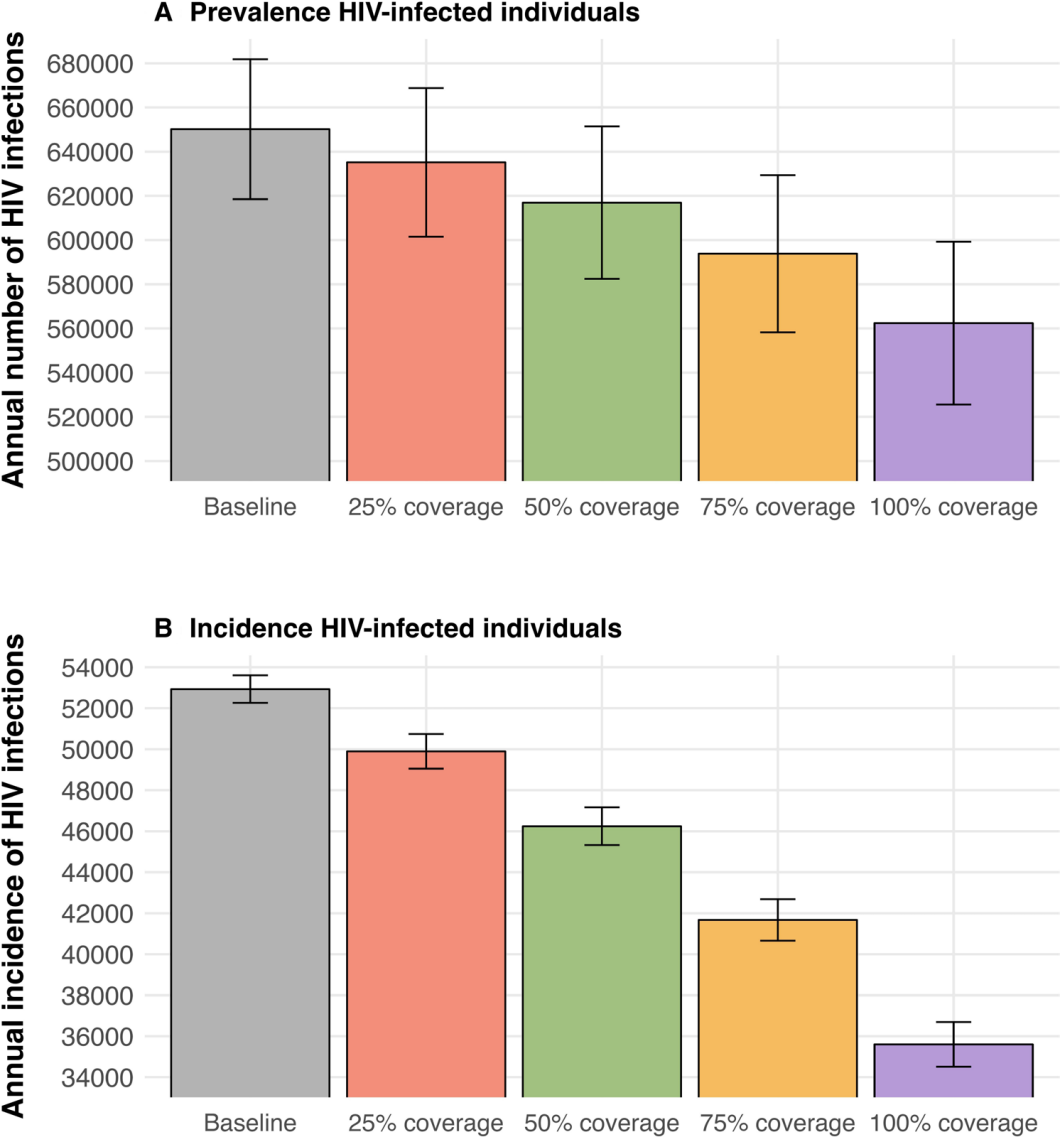
(**A**) Prevalence number of HIV-associated infections and (**B**) annual incidence of HIV infections by health insurance coverage scheme. CIs were computed based on Cameroon’s prevalence lower and upper bounds. Baseline means null health insurance coverage (0%).

Table 1 shows the main base-case results. Compared with a baseline scenario with zero health insurance coverage among WGTS, introducing a 25% coverage level results in higher incremental costs but improved efficiency, with an ICER_25%_ of £2795 per DALY averted (95% CI £2483 to £2824). Increasing coverage to 50%, 75% and 100% decreases the ICER per DALY averted by 9.1% (£2541; 95% CI £2370 to £2592), 18.9% (£2263; 95% CI £2156 to £2316) and 29.9% (£1952; 95% CI £1891 to £1998), respectively. These coverage levels correspond to averting thousands of infections (deaths): 751 (22), 1662 (51), 2818 (92) and 4338 (142) compared with no coverage. With these strategies, annual HIV prevalence could decrease by 2.2% under 25% coverage, 5.0% under 50%, 8.5% under 75% and 13.2% under full coverage, respectively (figure 2A). This results in a significant reduction in the annual incidence of HIV cases to 52 332, 49 532, 41 649 and 35 804 compared with 52 266 cases when no health insurance is provided (figure 2B). Online supplemental figure A3 displays annual HIV-associated deaths per strategy.

**Table 1:** Cost-effectiveness of health insurance among WGTS following different coverage schemes and over a 50-year time horizon (in 2023 £)

Health insurance coverage among WGTS	Total costs(in 10 000 £)	Incremental costs(in 10 000 £)	DALYs(in 1000 s)	DALYs averted(in 1000 s)	HIV infections(in 1000 s)	HIV infections averted(in 1000 s)	HIV deaths(in 1000 s)	HIV deaths averted(in 1000 s)	ICER (/DALYs averted)	ICER(/HIV infection averted)
0%	246 724 (230 369–262 470)		9791 (9004–10 555)		32 509 (30 925–33 961)		958 (906–1006)		–	–
25%	297 255 (280 326–313 082)	50 531 (50 612–49 956)	9611 (8800–10 379)	180 (176–205)	31 758 (30 076–33 303)	751 (658–849)	936 (879–984)	22 (22–27)	2795(2483–2824)	673(597–756)
50%	347 488 (330 402–363 432)	100 764 (100 962–100 033)	9395 (8577–10 169)	396 (386–428)	30 847 (29 121–32 427)	1662 (1534–1804)	907 (848–955)	51 (51–58)	2541(2370–2592)	606(561–652)
75%	397 165 (379 862–413 281)	150 441 (150 810–149 492)	9127 (8301–9907)	664 (648–703)	29 691 (27 912–31 315)	2818 (2646–3013)	866 (809–919)	92 (87–97)	2263(2156–2316)	534(502–566)
100%	445 732 (428 130–462, 093)	199 008 (199 622–197 760)	8772 (7940–9559)	1019 (996–1065)	28 121 (26 279–29 799)	4388 (4163–4646)	816 (758–870)	142 (136–149)	1952(1891–1998)	454(432–477)

Notes: -adjusted life years. cost-effectiveness ratio. ICERs are compared tocompared with the baseline scenario with 0% health insurance coverage. girls engaging in transactionalsex. Costs and DALYs were discounted at 3%.

DALYsdisability-adjusted life-yearsICERincremental cost-effectiveness ratioWGTSwomen and girls engaging in transactional sex

### Univariable and scenario analysis

Changes in the population size of WGTS had a negligible effect on the ICER (±1%), irrespective of the health insurance scheme (table 2). Adjusting health insurance costs by ±50% altered ICERs by 54%–55%, whereas a 100% effect of the health insurance in reducing HIV could decrease ICERs by 12.8%–20.9%, depending on coverage levels—with reductions diminishing to lesser extents at 80% effect levels. Altering discount rates to 6% led to an increase in ICERs/DALY averted by 43.6%–53.6%. Enhancing ART coverage to average population levels (≈79%) could potentially raise ICERs/DALY averted by up to 284.8% in scenarios of full coverage, mitigating the benefits of health insurance. Table 3 presents the costs and health outcomes of introducing PrEP among WGTS under various coverage scenarios, including a combination of PrEP and health insurance. PrEP alone proved more expensive and less cost-effective than health insurance alone, with ICERs per DALY averted ranging from £14 628 to £19 956, depending on coverage levels and if compared with a status quo scenario with 0% health insurance coverage. However, increasing PrEP coverage to 20% among WGTS, combined with 75%–100% health insurance, raises ICERs by 6.5%–6.6% compared with 75% insurance alone, achieving the lowest ICER per DALY or HIV infection averted when both strategies are implemented (table 3).

**Table 2:** Scenario and univariate analysis of the implementation of the health insurance among WGTS in Cameroon over 50 years (in 2023 £)

Variables	Health insurance coverage among WGTS	Incremental costs(in 10 000 s £)	DALYs averted(in 1000 s)	HIV infections averted(in 1000 s)	HIV deaths averted(in 1000 s)	ICER (/DALYs averted)	% var base case‡	ICER (/HIV infection averted)	% var base case‡
Increased population size among WGTS (≈3.36%)	0%*	246 294	9792	32 480	958	–	–	–	–
	25%	51 316	185	759	22	2772	−0.81%	676	0.49%
	50%	102 324	406	1680	52	2520	−0.84%	609	0.48%
	75%	152 761	681	2851	93	2244	−0.85%	536	0.35%
	100%	202 055	1045	4441	144	1934	−0.90%	455	0.22%
Decreased population size among WGTS (≈0.04%)	0%*	245 489	9703	32 321	951	–	–	–	–
	25%	48 437	173	731	21	2798	0.09%	663	−1.51%
	50%	96 585	380	1618	50	2543	0.08%	597	−1.48%
	75%	144 198	636	2743	89	2266	0.11%	526	−1.55%
	100%	190 737	976	4270	138	1954	0.11%	447	−1.61%
Increased health insurance costs (≈£120pp)	0%*	246 724	9791	32 509	958	–	–	–	–
	25%	77 757	181	751	22	4301	53.87%	1036	53.93%
	50%	155 482	397	1662	51	3920	54.28%	935	54.37%
	75%	233 007	665	2818	92	3505	54.90%	827	54.82%
	100%	309 938	1019	4388	142	3040	55.75%	706	55.57%
Decreased health insurance costs(≈£40pp)	0%*	246 724	9791	32 509	958	–	–	–	–
	25%	23 305	181	751	22	1289	−53.88%	310	−53.87%
	50%	46 046	397	1662	51	1161	−54.31%	277	−54.28%
	75%	67 876	665	2818	92	1021	−54.88%	241	−54.90%
	100%	88 079	1019	4388	142	864	−55.74%	201	−55.79%
Increased insurance effect in reducing HIV (≈100%)	0%*	246 716	9791	32 507	958	–	–	–	–
	25%	50 026	205	853	25	2437	−12.81%	586	−12.87%
	50%	99 567	457	1920	59	2179	−14.26%	519	−14.44%
	75%	148 166	783	3338	108	1892	−16.41%	444	−16.88%
	100%	194 689	1248	5420	174	1560	−20.09%	359	−20.89%
Decreased insurance effect in reducing HIV (≈80%)	0%*	246 724	9791	32 509	958	–	–	–	–
	25%	50 936	161	668	20	3161	13.10%	762	13.23%
	50%	101 693	349	1461	45	2910	14.52%	696	14.83%
	75%	152 120	576	2434	79	2640	16.64%	625	17.03%
	100%	201 917	862	3686	120	2342	20.00%	548	20.65%
Lower discount rate, ≈0%	0%*	528 346	25 147	32 509	958	–	–	–	–
	25%	118 754	461	668	20	2579	−7.74%	1777	164.00%
	50%	237 059	1000	1461	45	2370	−6.74%	1622	167.67%
	75%	354 508	1653	2434	79	2145	−5.23%	1456	172.73%
	100%	470 286	2479	3686	120	1897	−2.80%	1276	181.01%
Increased discount rate, ≈6%	0%*	139 897	4526	32 509	958	–	–	–	–
	25%	26 155	65	668	20	4013	43.58%	391	−41.86%
	50%	52 221	141	1461	45	3702	45.69%	357	−41.04%
	75%	78 131	232	2434	79	3367	48.79%	321	−39.89%
	100%	103 752	346	3686	120	2999	53.63%	281	−38.00%
Decreased ART coverage among WGTS (≈35%)	0%*	250 882	10 017	33 482	992	–	–	–	–
	25%	50 196	200	823	25	2512	−10.14%	610	−9.41%
	50%	100 020	439	1829	57	2278	−10.36%	547	−9.74%
	75%	149 164	737	3113	103	2023	−10.62%	479	−10.27%
	100%	196 958	1134	4873	160	1737	−11.02%	404	−10.96%
Increased ART coverage among WGTS (≈79%)†	0%*	216 697	8358	25 918	742	–	–	–	–
	25%	53 450	53	179	3	10 124	262.21%	2993	344.74%
	50%	107 095	115	386	9	9349	267.93%	2777	358.19%
	75%	160 967	190	634	18	8494	275.34%	2538	375.21%
	100%	215 109	286	950	27	7513	284.88%	2264	398.65%

Notes: -adjusted life years. cost-effectiveness ratio. ICERs are compared tocompared with the baseline scenario with 0% health insurance coverage.We reported the raw values for the coverage scenario ( no incremental costs or outcomes as the 25, 50, 75, and 100 coverage schemes depict). girls engaging in transactional sex. *We analysed if ART coverage among WGTS was same as the overall population.

* We reported the raw values for the 0% coverage scenario (ie, no incremental costs or outcomes as the 25, 50, 75 and 100 coverage schemes depict).

† We analysed if ART coverage among WGTS was the same as the overall population.

‡ Percent variation was calculated relative to baseline scenario with 0% health insurance coverage.

ARTantiretroviral therapyDALYsdisability-adjusted life-yearsICERincremental cost-effectiveness ratioWGTSwomen and girls engaging in transactional sex

**Table 3:** Scenario analysis of the implementation of different PrEP schemes and health insurance coverage among WGTS in Cameroon over 50 years (in 2023 £)

PrEP coverage schemes	Health insurance coverage among WGTS	Incremental costs(in 10 000 s £)	DALYs averted(in 1000 s)	HIV infections averted(in 1000 s)	HIV deaths averted(in 1000 s)	ICER (/DALYs averted)	% var base case*	ICER (/HIV infection averted)	% var base case*
PrEP coverage among WGTS (≈20%)	0%†	417 869	9674	31 893	939	14 628*	—	2778*	—
	25%	51 367	171	706	20	3003	7.43%	717	6.54%
	50%	102 599	376	1563	48	2732	7.54%	646	6.60%
	75%	153 519	630	2651	86	2436	7.65%	569	6.62%
	100%	203 715	969	4135	134	2102	7.67%	483	6.50%
PrEP coverage among WGTS (≈40%)	0%†	591 050	9604	31 593	929	18 413*	—	3759*	—
	25%	52 338	164	681	20	3194	14.27%	769	14.18%
	50%	104 685	358	1500	46	2923	15.04%	698	15.09%
	75%	156 947	597	2527	82	2628	16.10%	621	16.34%
	100%	208 897	909	3897	126	2297	17.66%	536	18.20%
PrEP among WGTS (≈60%)	0%†	765 439	9528	31 263	918	19 723*	—	4163*	—
	25%	53 268	155	651	19	3427	22.61%	819	21.63%
	50%	106 666	338	1426	44	3155	24.19%	748	23.40%
	75%	160 164	560	2383	77	2861	26.42%	672	25.92%
	100%	213 673	843	3629	117	2535	29.86%	589	29.85%
PrEP among WGTS (≈80%)	0%†	941 200	9443	30 898	906	19 956*	—	4311*	—
	25%	54 148	145	613	18	3724	33.23%	883	31.11%
	50%	108 519	314	1336	41	3452	35.85%	812	34.00%
	75%	163 129	516	2212	71	3159	39.57%	737	38.13%
	100%	217 980	768	3323	107	2838	45.37%	656	44.64%
PrEP among WGTS (≈100%)	0%†	1 118 540	9350	30 488	893	19 769*	—	4314*	—
	25%	54 963	133	567	16	4124	47.54%	969	43.93%
	50%	110 211	286	1226	37	3851	51.58%	899	48.32%
	75%	165 785	466	2009	65	3560	57.32%	825	54.63%
	100%	221 738	683	2971	95	3246	66.26%	746	64.55%

Notes: -adjusted life years. cost-effectiveness ratio. ICERs are compared tocompared with the baseline scenario with 0% health insurance coverage.We reported the raw values for the coverage scenario ( no incremental costs or outcomes as the 25, 50, 75, and 100 coverage schemes depict). girls engaging in transactional sex. These ICERs were computed using the health insurance scenario from Table 1 as a reference to evaluate the cost-effectiveness of PrEP only against a do-nothing scenario.

* These ICERs were computed using the 0% health insurance scenario from table 1 as a reference to evaluate the cost-effectiveness of PrEP only against a do-nothing scenario.

† We reported the raw values for the 0% coverage scenario (ie, no incremental costs or outcomes as the 25, 50, 75 and 100 coverage schemes depict).

DALYsdisability-adjusted life-yearsICERincremental cost-effectiveness ratioPrEPpre-exposure prophylaxisWGTSwomen and girls engaging in transactional sex

### Probability sensitivity analysis and WTP

In the PSA for the 25% health insurance coverage scenario, we observed incremental DALYs averted at 110 000 with corresponding incremental costs of £536.9 million, resulting in an average ICER of £4859 per DALY averted (online supplemental figure A4). For higher coverage levels—50%, 75% and 100%—the average ICERs were £3743, £2806 and £2128 per DALY averted, respectively. With a WTP threshold set at £1239 (Cameroon’s GDP per capita), cost-effectiveness ranged between 50% and 58% across all coverage levels if aiming to avert DALYs (online supplemental figure A4), peaking at 100% coverage (greater benefits). Online supplemental figure A5 displays PSA over ICER/infection averted exhibiting more substantial efficiencies than for DALYs, with 80% simulations<WTP at 100% coverage.

### Most influential parameters and bivariable link between health insurance and its effect in reducing HIV prevalence

Online supplemental figure A6, panel A, highlights the most influential parameters in our model. An increase in the frequency of casual sex partners correlates with a rise in HIV infections (PRCC=0.34, p=0.003). Conversely, parameters that negatively influence HIV infections demonstrate significant impacts. These include the efficacy of ART in preventing HIV (PRCC=−0.62, p<0.001), the proportion of low-risk females (PRCC=−0.33, p<0.001) and the percentage of low-risk men and HIV-positive women on ART in the general population (PRCC=−0.31, p<0.001 and PRCC=−0.24, p=0.009, respectively). Online supplemental figure A5, panel B, illustrates how enhancing health insurance effect in reducing HIV and expanding coverage among WGTS significantly lowers the annual HIV infection rate. Replacing a 0% coverage scenario with 100% effect/coverage leads to a reduction in infections from 640 000 to 540 000—an average decrease of 18.5%. Sustaining health insurance effect in reducing HIV and coverage at 70% or higher yields the most substantial health benefits, achieving a 10% reduction in total HIV infections compared with scenarios with no health insurance among WGTS. Similar results are found for HIV-associated deaths (online supplemental figure A7).

## Discussion

Our findings illustrate the pivotal role of health insurance in reducing the burden of HIV among WGTS and in the general population. Incremental increases in health insurance coverage, from 25% to 100%, consistently reduced ICERs per DALY averted, demonstrating enhanced cost-effectiveness with broader coverage.

Recent studies have shown the cost-effectiveness of HIV interventions for FSW, with costs ranging from £13^28^ to £1442^29^ for DALYs averted and £17^30^ to £2799^31^ for HIV infections averted. Average costs per participant have been reported up to £198,^32 33^ underscoring the economic feasibility of expanding HIV prevention to WGTS, particularly in high HIV prevalence settings.^32 34^ Most studies focus on biomedical (testing, condom distribution), behavioural (counselling) and structural interventions (microfinance, vouchers) targeting FSWs alone.^35^ Our study contributes to the literature by evaluating a national-scale intervention that incorporated consultations and covered pharmaceutical, medical and hospitalisation fees for non-chronic pathologies, extending benefits to up to six economic dependents of each WGTS.^19^ This inclusive approach resulted in higher ICERs due to increased per-person costs annually. However, it is important to note that studies on interventions such as circumcision and PrEP report a higher cost range per infection averted than the ones we obtained, from £4794^36 37^ to £41 167.^38^ This finding reflects the variability in intervention types, designs and contexts, especially when mixed designs are adopted by inclusion of family members/partners with multibenefit designs.^39^

The sensitivity analyses underscored the robustness of ICER to changes in the population size of WGTS and highlighted the sensitivity of cost-effectiveness to fluctuations in health insurance costs and efficiencies. Notably, the optimal health insurance effect in reducing HIV prevalence—particularly at or above 70%—emerged as crucial for maximising health gains, significantly reducing the annual incidence of HIV. This suggests that strategies focused on enhancing the quality and comprehensiveness of the health insurance package could be more impactful than changes in demographic parameters alone. Our PSA further supports these conclusions, with higher coverage scenarios consistently surpassing the WTP threshold derived from Cameroon’s GDP per capita. This indicates that increasing health insurance coverage is a cost-effective strategy, even when considering varying economic thresholds. We found that the most influential parameters identified—such as the frequency of casual sex partners and the effectiveness of ART—highlight the complex interplay between behavioural factors^40^,^42^ and treatment efficacy^43 44^ in shaping HIV epidemiology among WGTS. Also, the negative association of increased ART coverage with HIV infections reinforces the importance of integrating comprehensive ART programmes within health insurance frameworks to maximise public health outcomes, as prior studies did in Cameroon.^45 46^ One study found that 38% of patients did not adhere to their ART in Cameroon,^45^ and 53.9% have missed appointments for ART services. These findings could suggest that the high efficacy rates (ie, 96%) and coverage estimates (approximately 79% among populations other than WGTS) used in our model may be overly conservative. Lower actual ART efficacy or coverage would result in lower ICERs per DALYs/infections averted in our model, as more individuals would remain vulnerable to HIV, thereby increasing the relative impact of the health insurance scheme in reducing HIV transmission rates. However, if implemented, it is also vital to monitor the effectiveness of the health insurance policy over time.^47 48^

Significant differences emerged between the cost-effectiveness health insurance scheme and PrEP when analysed separately. The PrEP scheme demands significantly higher investment across all coverage levels. As exploratory budget analyses (online supplemental table A5), the hypothetical government expenditure for the PrEP scheme would rise steeply with coverage, from £86.85 millions (6.1% of annual health expenditure) at 25% to £347.4 millions (24.5%) at full coverage, while the health insurance scheme is less costly, ranging from £20.50 millions (1.4%) to £81.98 millions (5.8%). Based on the POWER study, PrEP was found to be a more expensive alternative and less cost-effective; therefore, balancing lower coverage of PrEP with higher coverage of a health insurance scheme would be an optimal strategy.

Our study has limitations. Most significantly, the parameter inputs derived from the literature may not accurately reflect the local characteristics of the study population, potentially limiting their generalisability in settings with scarce data. Nevertheless, we incorporated the most recent local studies, including the POWER randomised controlled trial, and conducted comprehensive sensitivity analyses to minimise biases and parameter uncertainty effects. Additionally, approximately 20%^49^ of individuals are unaware of their HIV status in Cameroon, which could lead to an underestimation of the actual impact of the health insurance scheme on infections/DALYs averted. Our analysis did not differentiate outcomes by age and excluded other UNAIDS high-risk populations, such as MSM, injecting drug users and transgender individuals, primarily due to data constraints. These groups could influence overall HIV transmission rates.^1 50^ An additional consideration is moral hazard in transactional sex should such an intervention be scaled up. While there was no evidence of within-study ex ante and ex post moral hazard,^19^ there could be an increase in women and girls engaging in such relationships, raising overall aggregate risk and transmission that might hamper impacts. With no national data on WGTS populations available, we relied on FSW estimates,^23^ which may bias the true impact of interventions. However, sensitivity analyses showed minimal effects when varying WGTS population sizes. Finally, while identifying WGTS for eligibility in such a health scheme presents challenges^51^—an aspect not covered in this study—we recognise the trade-off between inclusivity and efficiency. A broader approach may reduce stigma and improve access but risks diluting effectiveness by including those with lower relative benefit. A more targeted strategy could optimise resource allocation but must be balanced against feasibility, outreach and acceptability challenges. Transactional sex is particularly common among high school adolescent girls and university students, positioning them as a high-risk group for targeted interventions. Framing such programmes within broader, non-stigmatising categories—such as adolescent sexual health or student wellness initiatives—can facilitate identification and outreach while maintaining cultural and social sensitivity, making the programme easier to scale.^51^

However, despite this, we believe the full benefits of the health insurance scheme are likely underestimated, as it does not consider wider societal advantages (positive externalities) like enhanced family health and lower rates of other communicable diseases, which could have further implications for various health outcomes. Additional economic spill-overs not captured in this study, such as higher productivity (in school or work). Indeed, other work evaluating secondary outcomes of the POWER^19^ study suggest that forgone household medical spending is being spent on children’s education. The findings of this study would be generalisable to similar low-resource Africa settings where there are large gender disparities in HIV and transactional sex is prevalent such as in eastern and southern Africa. Free health insurance will also support progress towards achieving Sustainable Development Goal 3.8 by advancing universal health coverage on Sustainable Development Goal 3.3 in Cameroon.

This research demonstrates the cost-effectiveness of health insurance and potentially other highly effective structural interventions targeting WGTS, who receive minimal HIV prevention services and are excluded from key programmes like PrEP as they are not considered a ‘key population’.^52 53^ Our study targets social determinants of health and inequality among high-risk populations in Cameroon; essential for HIV prevention.^54^ Our findings align with WHO recommendations, suggesting that programmes for HIV prevention should be holistic and complementary.^55^ This is crucial due to the inseparable link between gender, political-legal and economic structures and the vulnerability of WGTS to HIV.^56^ By integrating health insurance with comprehensive ART and PrEP coverage, policy-makers can significantly reduce HIV transmission and enhance health outcomes. This approach strengthens public health infrastructure in high-burden and low public resource countries like Cameroon and promotes efficient resource allocation,^57^ providing a viable path towards the WHO’s 95-95-95 HIV targets.^58 59^

## supplementary material

10.1136/bmjgh-2024-017870 online supplemental file 1

## Data Availability

Data are available in a public, open access repository. All data relevant to the study are included in the article or uploaded as supplementary information.
