# Supplementary material for: Cost-effectiveness of health insurance among women engaged in transactional sex and impacts on HIV transmission in Cameroon: a mathematical model
Source: BMJ Glob Health. 2025 Feb 18;10(2):e017870. doi: 10.1136/bmjgh-2024-017870 (PMC11836847; doi:10.1136/bmjgh-2024-017870)
Supplement: online supplemental file 1 [file bmjgh-10-2-s001.pdf]

Supplementary appendix

Cost-effectiveness of health insurance among women engaged in transactional sex and impacts on HIV transmission in Cameroon: A Mathematical Model

Table of Contents

Figure A1. General schematics between WGTS and their sugar daddies 2

Figure A1.1 General schematics between WGTS and their sugar daddies 3

Figure A2. Schematics between WGTS, sugar daddies, and low-risk females and males 4

Text A1. Model description 5

Table A1. Baseline conditions 6

Table A2. Summary of main parameters among WGTS and their sugar daddies 7

Table A3. Model parameters 8

Text A2. Differential Equations 11

Table A4. Adult HIV prevalence in Cameroon over time 14

Figure A3. Annual number of HIV-associated deaths by health insurance coverage. 15

Figure A4. Probability sensitivity analysis of incremental costs and DALYs and willingness-to-pay thresholds using different health insurance schemes among WGTS over 50 years 16

Figure A5. Probability sensitivity analysis of incremental costs and HIV infections and willingness-to-pay thresholds using different health insurance schemes among WGTSs over 50 years 17

Figure A6. (A) Global sensitivity analysis with most influential parameters over prevalence of HIV infected individuals and (B) Dual sensitivity analysis between health insurance coverage and efficiency over annual number of people HIV-infected. 18

Figure A7. Sensitivity analyses for the annual number of people who died due to HIV by health insurance coverage and efficiency levels. 19

Table A5. PreP and Health insurance spending regarding hypothetical national expenditure considering our modelled population of WGTS and using different coverage levels (if administered separately) 20

Reflexivity statement -summary- 21

Reflexivity Statement questions following BMJ Global Health guidelines 22

References 24

Figure A1. General schematics between WGTS and their sugar daddies

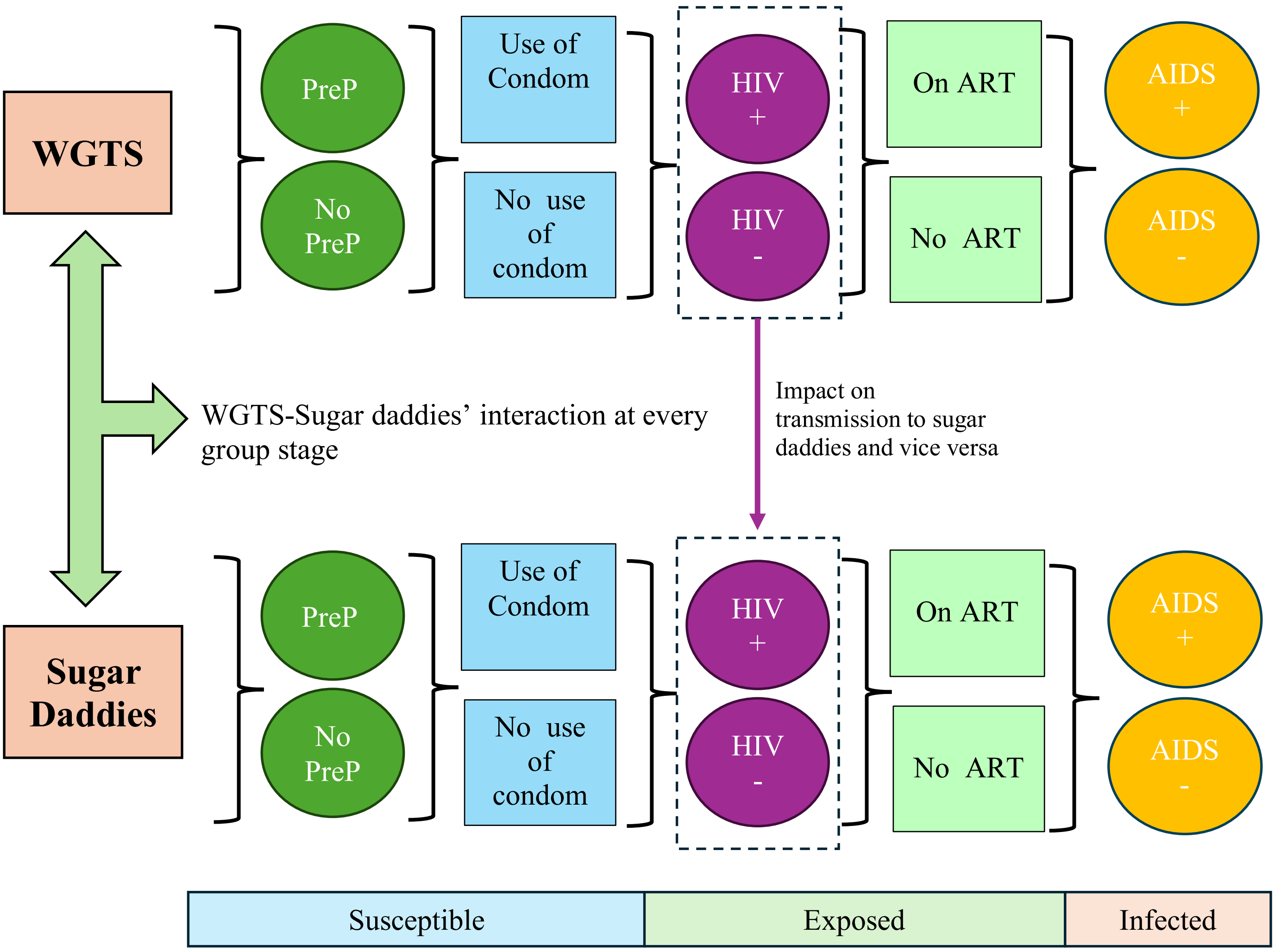

Notes: WGTS= Women and girls engaging in transactional sex (or sugar baby). PreP= Pre-exposure prophylaxis. HIV= Human immunodeficiency virus. ART= Antiretroviral therapy. AIDs= acquired immunodeficiency syndrome.

Figure A1.1 General schematics between WGTS and their sugar daddies

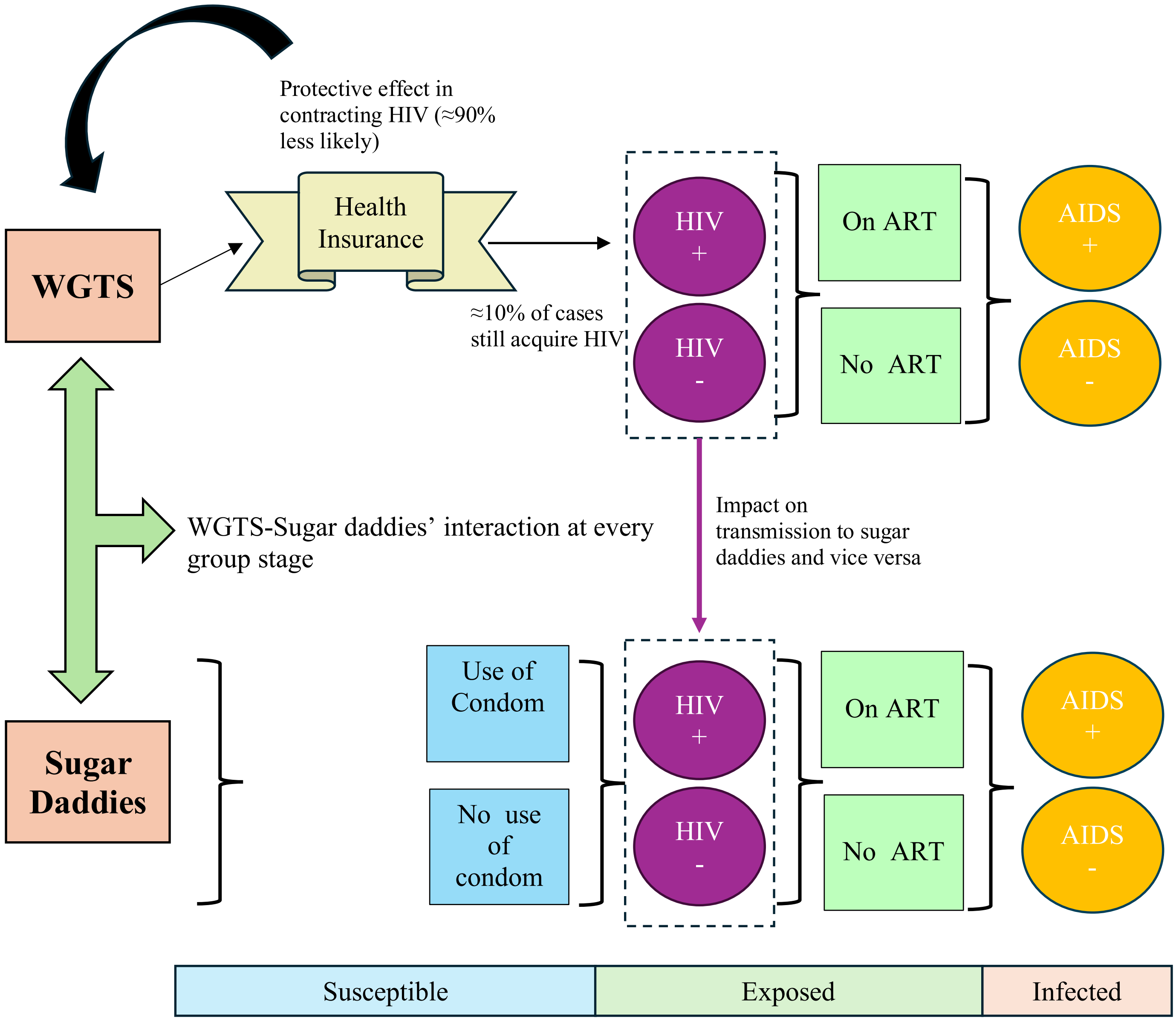

Notes: WGTS= Women and girls engaging in transactional sex (or sugar baby). PreP= Pre-exposure prophylaxis. HIV= Human immunodeficiency virus. ART= Antiretroviral therapy. AIDs= acquired immunodeficiency syndrome.

Figure A2. Schematics between WGTS, sugar daddies, and low-risk females and males

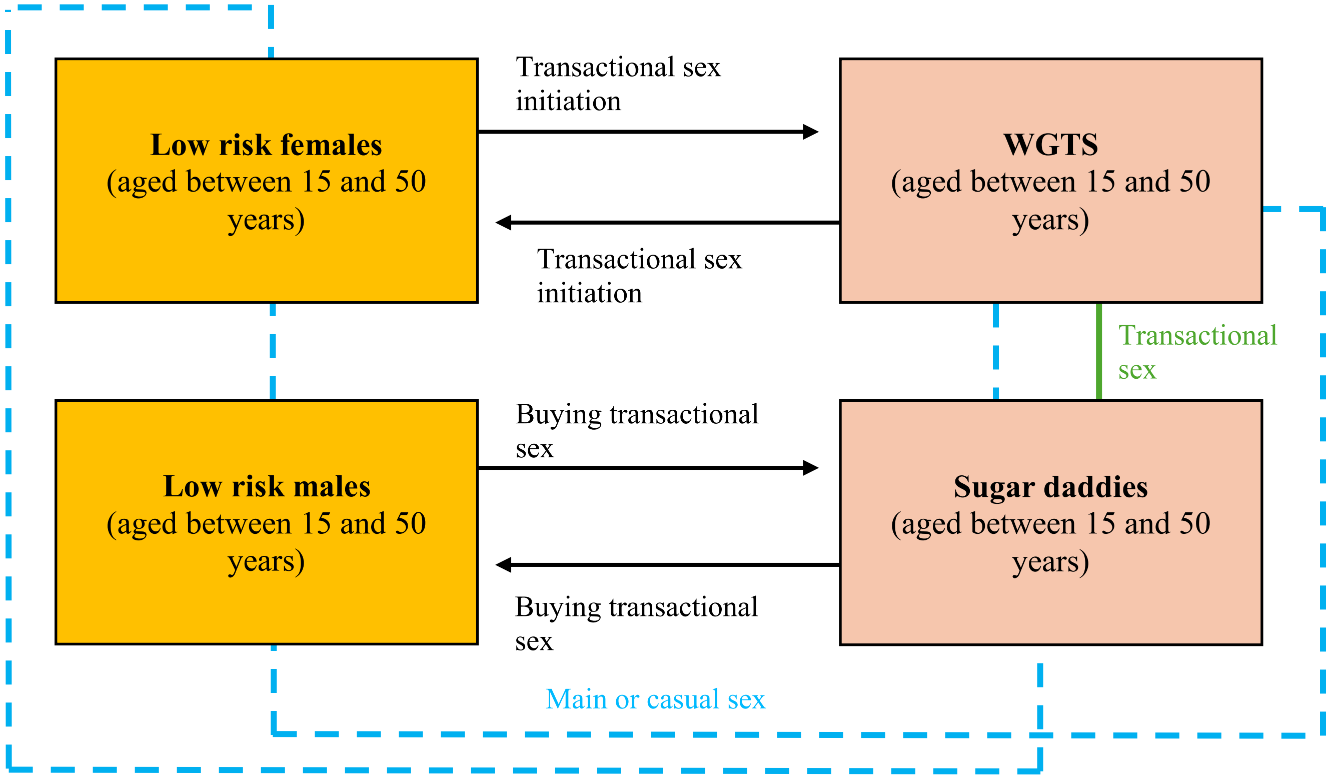

Notes: Ageing (<50 years of age) occurs at every single stage. WGTS= Women and girls engaging in transactional sex.

Text A1. Model description

We used a previously published dynamic HIV transmission model[1,2]. The model considers adults (>15 years of age) and divides the population into four sub-populations: low-risk females (i=1) and males (i=2), sugar daddies (i=3), and WGTS (i=4). Low-risk individuals are defined as people that do not report commercial sex. People enter the modelled population when they become sexually active, at a rate θ that balances non-HIV deaths and reflects population growth (with μ_1 as natural mortality rate in the country), with a proportion p entering the low-risk female group, (1-p) entering the low-risk male group. Low-risk females and males enter the model as HIV-negative. Low-risk females become WGTS at a rate κ and stay as WGTS for duration $1/\gamma$ years. Similarly, sugar daddies transition from the low-risk male population at rate z.

Sugar daddies remain as sugar daddies for a period of 1/g years before returning to the low-risk male group.

The model stratifies the population with respect to HIV infection and disease progression such that for each sub-population i, there is uninfected (S_i), acute infection (E_i), chronic infection (I_i) and deceased (R_i). Upon infection (β= transmission parameter derived from sexual intercourses), susceptible individuals move to the acute stage of HIV infection before progressing to the chronic, pre-AIDS and AIDS phases of infection at fixed rates ($\eta_1$for acute to AIDS with no ART treatment, $\eta_2$for acute to AIDS on ART treatment). Individuals with AIDS experience a HIV-related mortality rate δ_2 if on ART, and it is higher if off ART δ_1. All sub-populations also experience non-HIV related death and exit the model with rates μ_i, which incorporates both non-HIV related death rates and ageing out of the model. The model incorporates HIV transmission due to main, casual and commercial sexual partnerships. Commercial partnerships can only occur between WGTS and their sugar daddies. The risk of HIV transmission for a particular individual is related to the HIV prevalence of their sexual partners. Transmission risk is also related to the average frequency of sex acts (denoted by ${\Psi_{ij}}^{k}$) for different types of partnerships and between risk groups i and j, where k denotes the type of sexual partner (main, casual or commercial) through vaginal sex and i=population type and j= population to have sex with. HIV transmission is reduced through condom use by a factor $\left(1-\varepsilon\pi_{ij}^{k}\right)$, where ε is the efficacy of condom use and π_{ij}^{k} is the average consistency of condom use reported by those in risk groups i and j. The consistency of condom use varies depending on the type of partnership. HIV susceptible populations can initiate PrEP at a time varying rate (ν_i), and PreP has an efficiency of e_ν. Low-risk populations are assumed to have ν_i=0, and WGTSs has a 0% coverage as reported in the POWER study.[3] A proportion (α_i and τ_j) of HIV-infected acute and AIDS populations initiate ART, which is lower among WGTS. ART treatment has an efficacy on preventing HIV transmission equal to $e_\alpha = e_\tau$.

Table A1. Baseline conditions

Parameter/variable	Value	Source
Prevalence at endemic level P_i^{∞}	i=1; 2.7%<br>i=2; 2.7%<br>i=3; 3.0%<br>i=4; 3.4%	Literature among Cameroon[4] population and POWER[3]
Total adult population Cameroon (15-64 years) '*tpop*'	27,914,536*0.50	Cameroon's total population * % population aged 15-64. World Bank[5,6]
Percentage of female population (*pfem*)	51%	World Bank[7]
Percentage of male population (*pmale*)	(100% -51%)	Assumed
Percentage of male sugar daddies (*pcli*)	14% of male population	Literature[8]
Percentage of female WGTS (*pWGTS*)	1.96%	Literature[9]
Male population (*mpop*)	51%**tpop*	Calculated
Female population (*fpop*)	(100%-51%)**tpop*	Calculated
Proportion of HIV that developed AIDS (%) and are alive (*AIDsp*)	6.3%	Calculated as an average between η_1 and η_2 (see Table A3)
Baseline conditions		
Low risk female HIV-susceptible S_1	*fpop**(1-*pWGTS*)*(1-$P_{i=1}^{\infty}$)	Calculated
Low risk female HIV-infected acute E_1	*fpop**(1-*pWGTS*)*($P_{i=1}^{\infty}$) *(1- *AIDsp*)	Calculated
Low risk female AIDS I_1	*fpop**(1-*pWGTS*)*($P_{i=1}^{\infty}$) *(*AIDsp*)	Calculated
Low risk male HIV-susceptible S_2	*mpop**(1-*pcli*)*(1-$P_{i=2}^{\infty}$)	Calculated
Low risk male HIV-infected acute E_2	*mpop**(1-*pcli*)*($P_{i=2}^{\infty}$) *(1- *AIDsp*)	Calculated
Low risk male AIDS I_2	*mpop**(1-*pcli*)*($P_{i=2}^{\infty}$) *(*AIDsp*)	Calculated
Sugar daddies HIV-susceptible S_3	*mpop**(*pcli*)*(1-$P_{i=3}^{\infty}$)	Calculated
Sugar daddies HIV-infected acute E_3	*mpop**(*pcli*)*(1-$P_{i=3}^{\infty}$)* (1- *AIDsp*)	Calculated
Sugar daddies AIDS I_3	*mpop**(*pcli*)*(1-$P_{i=3}^{\infty}$)* (*AIDsp*)	Calculated
WGTS HIV-susceptible S_4	*fpop**(*pWGTS*)*(1-$P_{i=4}^{\infty}$)	Calculated<br>Calculated
WGTS HIV-infected acute E_4	*fpop**(*pWGTS*)*(1-$P_{i=4}^{\infty}$)* (1- *AIDsp*)	
WGTS AIDS I_4	*fpop**(*pWGTS*)*(1-$P_{i=4}^{\infty}$)* (*AIDsp*)	Calculated
Dead individuals (R_1, R_2, R_4, R_4)	0	Assumed

Notes: WGTS= Women and girls engaging in transactional sex.

Table A2. Summary of main parameters among WGTS and their sugar daddies

Parameter	WGTS (Sugar babies)		Sugar daddies	
	Value	Source	Value	Source
Population size (% total)	1.96%	Literature[9]	Balance transactional sex acts	Assumed
HIV prevalence	3.4%	POWER[3]	3.0%	Literature[10]
Average life duration of Transactional sex (months)	38 months	POWER[3]	38 months	Assumed
Number of sugar daddies or WGTS (sugar daddies or babies, respectively) in a typical week	2.7	POWER[3]	2.7	Assumed.
Number of sex acts in a typical week	Depends on subpopulation; see supplementary material, Table A1-3.			
Use of condom last sex act	50%	POWER[3]	50%	Assumed.
Efficiency of health insurance impact on HIV	0.89 (0.82-1.00)	POWER[3]	NA	NA
ART coverage among WGTS living with HIV with supressed viral loads	41.7% (35.4-48.2)	Literarure[11]	79%	Literature[12]
Proportion of susceptible individuals using PrEP	0%	POWER[3]	0%	Assumed

Parameter	General	
	Value	Source
Effectiveness of using condom to prevent HIV	90%	Literature[13]
Effectiveness of ART treatment in preventing HIV transmission	96%	Literature[14]
Progression from HIV to AIDs if no ART	10-11 years	Literature[15]
Effectiveness of PreP in reducing HIV transmission in high-risk populations	51%	Literature[16]
Mortality rate attributed to HIV/AIDs on ART (annual)	16.3%	Literature[17]
Mortality rate attributed to AIDs off ART (annual)	23.5%	Literature[18]
**Unit costs (in 2022 £)**		
Cost of health insurance per year per person	£80	POWER[3]
Cost of PrEP per year per person	£339	Literature[19]
ART cost per year per person	£205-£672	Literature[20]
**DALYs**		
Acute/chronic HIV-AIDs infection, on ART	0.078 (0.05–0.11)	Literature[21]
Pre-AIDs, off ART	0.274 (0.18–0.38)	Literature[21]
AIDs, off ART	0.582 (0.41–0.74)	Literature[21]

Notes: POWER= Protecting women from economic shocks to fight HIV in Africa RCT (randomized controlled trial). WGTS= Women and girls engaging in transactional sex. NA= Not applicable. ART= Antiretroviral therapy. PrEP=Pre-exposure prophylaxis. *Health insurance cost per year per WGTS insured.

Table A3. Model parameters

Parameter/variable	Value	Source
Proportion of individuals who enter to the model as low risk female (p)	50.1%	World Bank[7]
Birth rate (θ)	0.035	World Bank[22]
Time that WGTS remain as sexual workers (1/γ)	γ=6 years (±4)	Literature[23]
Rate of low-risk females becoming WGTS (κ)	0.02	Literature[24]
Rate of non-HIV related death among male or female populations (μ_1)	0.009	World Bank[25]
Progression rate from HIV infection to AIDS, with no ART (η_1)	1/10; (10 years)	UNAIDs[26]
Progression rate from HIV infection to AIDS, on ART (η_2)	1/40 (40 years)	Literature[27]
Mortality rate related to AIDS, off treatment (δ_1)	23.5% (annually)	Literature[18]
Mortality rate related to AIDS on treatment (ART) (δ_2)	16.3% (annually)	Literature[17]
Proportion of low-risk male population becoming sugar daddies (z)	1%	Assumed
Time of that sugar daddies remain as sugar daddies ($1/g$)	3.2 years or 38 months	Assumed to balance populations
Transmission rate (β)	0.0028	Calibrated to HIV prevalence data[4] and model scheme.
Efficacy of condom use (ε)	90%	Literature[13]
Consistency of condom use reported (π_{ij}^{k})	$\boldsymbol{i=1, j=2, k=m}$ 33.65%	Literature [1]
	$\boldsymbol{i=1, j=2, k=c}$ 59.2%	Literature [1]
	$\boldsymbol{i=1, j=3, k=m}$ 33.65%	Literature [1]
		Literature [1]

	$i = 1, j = 3, k = c$<br>59.2%	
	$i = 2, j = 4, k = m$<br>33.65%	Literature [1]
	$i = 2, j = 4, k = c$<br>59.2%	Literature [1]
	$i = 3, j = 4, k = m$<br>33.65%	Literature [1]
	$i = 3, j = 4, k = c$<br>59.2%	Literature [1]
	$i = 3, j = 4, k = co$<br>50%	POWER[3]
Average frequency of sex acts (Ψ_{ij}^{k})	$i = 1, j = 2, k = m$<br>6-144 (per year);<br>Median = 75	Literature[28,29]
	$i = 1, j = 2, k = c$<br>0.5-10.2; Median = 5.2	Literature [1]
	$i = 1, j = 3, k = m$<br>6-144 (per year);<br>Median = 75	Literature[28,29]
	$i = 1, j = 3, k = c$<br>0.5-10.2; Median= 5.4	Literature [1]
	$i = 2, j = 4, k = m$<br>24-144 (per year);<br>Median= 84	Literature [30]
	$i = 2, j = 4, k = c$<br>0.2-8.3 (per year);<br>Median=4.3	Literature[28,29]
	$i = 3, j = 4, k = m$<br>6-144 (per year); Median=75	Literature [30]
	$i = 3, j = 4, k = c$<br>0.5-10.2; Median= 5.2	Literature [1]
	$i = 3, j = 4, k = co$<br>3.9 per week; 203 per year	POWER[3]
Proportion of population on PrEP (ν_i)	i=1; 0%<br>i=2; 0%<br>i=3; 0%<br>i=4; 0%	Assumed<br>Assumed<br>Assumed<br>POWER[3]
Efficiency of PrEP (e_ν)	51%	Literature[31]

Proportion of HIV on ART (α_i)	i=1; 79%<br>i=2; 79%<br>i=3; 79%<br>i=4; 41.7%	Literature[32]
Efficiency of ART to prevent HIV infection (e_α)	96%	Literature[14]
Frequency of sex partners (n_i^k)	$\boldsymbol{i=1, k=m}$<br>1-2 per year<br><br>$\boldsymbol{i=1, k=c}$<br>1.1-15.1 per year; Median=8.1<br><br>$\boldsymbol{i=2, k=m}$<br>1-2 per year; Median=<br><br>$\boldsymbol{i=2, k=c}$<br>1.1-15.1 per year<br><br>$\boldsymbol{i=3, k=m}$<br>1-2.9 per year<br><br>$\boldsymbol{i=3, k=c}$<br>1.1-15.1 per year<br><br>$\boldsymbol{i=3, k=co}$<br>2.3-72.8 per year<br><br>$\boldsymbol{i=4, k=m}$<br>1-3.1 per year<br><br>$i=\boldsymbol{4, k=c}$<br>1-18 per year<br><br>$\boldsymbol{i=4, k=co}$<br>2.7 per week, 141 per year	Assumed<br><br>Literature[1]<br><br>Assumed<br><br>Literature[1]<br><br>Literature[1]<br><br>Literature[1]<br><br>Literature[28,29]<br><br>Literature[28,29]<br><br>Literature[1,28,29]<br><br>POWER[3]
Proportion of WGTS with health insurance (s)	Adjusted to different values between 0 and 1.	Assumed.
Efficacy of health insurance in reducing HIV (e_s)	0.89	POWER[3]
Proportions of AIDS on treatment (ART) (τ_j)	i=1; 79%<br>i=2; 79%<br>i=3; 79%<br>i=4; 41.7%	Literarure[11,12]
Efficiency of AIDS treatment to prevent HIV transmission (e_τ)	0.96	Assumed to be equal to e_α (ART)

Notes: PreP= Pre-exposure prophylaxis. HIV= Human immunodeficiency virus. ART= Antiretroviral therapy. AIDs= acquired immunodeficiency syndrome. WGTS= Women and girls engaging in transactional sex.

Text A2. Differential Equations

Where m, c, and co stand for main, causal and commercial sex. t=time.

Low risk female population (i=1)

$$\frac{dS_1}{dt} = (1-\phi_1)p\theta N + \gamma S_4 - (\Lambda_1^m + \Lambda_1^c)S_1 - (\kappa + \mu_1)S_1$$

$$\frac{dE_1}{dt} = (\Lambda_1^m + \Lambda_1^c)S_1 + \gamma E_4 - ((1-\alpha_1)\eta_1 + \alpha_1\eta_2 + \kappa + \mu_1)E_1$$

$$\frac{dI_1}{dt} = \phi_1 p\theta + ((1-\alpha_1)\eta_1 + \alpha_1\eta_2)E_1 + \gamma I_4 - \big((1-\tau_1)\delta_1 + \tau_1\delta_2 + \kappa + \mu_1\big)I_1$$

$$\frac{dR_1}{dt} = ((1-\tau_1)\delta_1 + \tau_1\delta_2)I_1$$

Low risk male population (i=2)

$$\frac{dS_2}{dt} = (1-\phi_2)(1-p)\theta N + gS_3 - (\Lambda_2^m + \Lambda_2^c)S_2 - (z + \mu_2)S_2$$

$$\frac{dE_2}{dt} = (\Lambda_2^m + \Lambda_2^c)S_2 + gE_3 - ((1-\alpha_2)\eta_1 + \alpha_2\eta_2 + z + \mu_2)E_2$$

$$\frac{dI_2}{dt} = \phi_2(1-p)\theta + gI_3 + ((1-\alpha_2)\eta_1 + \alpha_2\eta_2)E_2 - \big((1-\tau_2)\delta_1 + \tau_2\delta_2 + z + \mu_2\big)I_2$$

$$\frac{dR_2}{dt} = ((1-\tau_2)\delta_1 + \tau_2\delta_2)I_2$$

Sugar daddies population (i=3)

$$\frac{dS_3}{dt} = zS_2 - (\Lambda_3^m + \Lambda_3^c + \Lambda_3^{co})S_3 - (g + \mu_3)S_3$$

$$\frac{dE_3}{dt} = (\Lambda_3^m + \Lambda_3^c + \Lambda_3^{co})S_3 + zE_2 - ((1-\alpha_3)\eta_1 + \alpha_3\eta_2 + g + \mu_3)E_3$$

$$\frac{dI_3}{dt} = ((1-\alpha_3)\eta_1 + \alpha_3\eta_2)E_3 + zI_2 - \big((1-\tau_3)\delta_1 + \tau_3\delta_2 + g + \mu_3\big)I_3$$

$$\frac{dR_3}{dt} = ((1-\tau_3)\delta_1 + \tau_3\delta_2)I_3$$

WGTS population (i=4)

$$\frac{dS_4}{dt} = \kappa S_1 - [1 - se_s][(\Lambda_4^m + \Lambda_4^c + \Lambda_4^{co})S_4 - (\gamma + \mu_4)S_4$$

$$\frac{dE_4}{dt} = [1 - se_s][(\Lambda_4^m + \Lambda_4^c + \Lambda_4^{co})S_4 + \kappa E_1 - ((1 - \alpha_4)\eta_1 + \alpha_4\eta_2 + \gamma + \mu_4)E_4$$

$$\frac{dI_4}{dt} = ((1 - \alpha_4)\eta_1 + \alpha_4\eta_2)E_4 + \kappa I_1 - \big((1 - \tau_4)\delta_1 + \tau_4\delta_2 + \gamma + \mu_4\big)I_4$$

$$\frac{dR_4}{dt} = ((1 - \tau_4)\delta_1 + \tau_4\delta_2)I_4$$

Force of Infection (FOI)

1. Low-risk female population (i=1)

$$\Lambda_1^k = \lambda_1 \frac{\rho_1^k}{N_1}[1 - \nu_1 e_v] \sum_{j=2,3} \beta(1 - \varepsilon\,\pi_{1j}^k)\Psi_{1j}^k n_j^k N_j B_j$$

2. Low-risk male population (i=2)

$$\Lambda_2^k = \lambda_2 n_2^k[1 - \nu_2 e_v] \sum_{j=1,4} \beta(1 - \varepsilon\,\pi_{2j}^k)\Psi_{2j}^k \rho_j^k B_j$$

3. Sugar daddies Population (i=3)

$$\Lambda_3^k = \lambda_3 n_3^k[1 - \nu_3 e_v] \sum_{j=1,4} \beta(1 - \varepsilon\,\pi_{3j}^k)\Psi_{3j}^k \rho_j^k B_j$$

$$\Lambda_3^{co} = \lambda_3 n_3^{co}\beta(1 - \varepsilon\pi_{34}^{co})B_4$$

4. WGTS (i=4)

$$\Lambda_4^k = \lambda_4 \frac{\rho_4^k}{N_4}[1 - \nu_4 e_v] \sum_{j=2,3} \beta(1 - \varepsilon\,\pi_{4j}^k)\Psi_{4j}^k n_j^k N_j B_j$$

$$\Lambda_4^{co} = \lambda_4 \Psi_{34}^{co}[1 - \nu_4 e_v](1 - \varepsilon\pi_{43}^{co})B_3$$

Where:

$$B_j = \frac{\left(1 - \alpha_j\, e_\alpha\right)E_j + (1 - \tau_j e_\tau)I_j}{N_j}$$

$$\lambda_i = we^{\frac{P_i(t)}{P_i^\infty}\ln\frac{1}{w}}$$

Where $P_i(t)$ is prevalence of HIV in population group i at time t and P_i^∞ is the prevalence at endemic level in that population group.

Probabilities p_j^k:

Probability of mixing with **low-risk females** for main and casual partnership	$\rho_1^k = \frac{n_1^k N_1}{n_1^k N_1 + n_4^k N_4}$
Probability of mixing with **low-risk males** for main and casual partnership	$\rho_2^k = \frac{n_2^k N_2}{n_2^k N_2 + n_3^k N_3}$
Probability of mixing with **sugar daddies of WGTS** for main and casual partnership	$\rho_3^k = \frac{n_3^k N_3}{n_2^k N_2 + n_3^k N_3}$
Probability of mixing with **WGTS** for main and casual partnership	$\rho_4^k = \frac{n_4^k N_4}{n_1^k N_1 + n_4^k N_4}$

These mixing equations assume that all sexual mixing casual and main partnerships is random based on the total number of sexual partnerships that each group provides.

Table A4. Adult HIV prevalence in Cameroon over time

Year	Adult HIV prevalence (%)
2001	5.0
2002	5.0
2003	5.0
2004	4.9
2005	4.8
2006	4.7
2007	4.6
2008	4.5
2009	4.4
2010	4.3
2011	4.1
2012	4.0
2013	3.9
2014	3.7
2015	3.6
2016	3.4
2017	3.3
2018	3.2
2019	3.0
2020	2.9
2021	2.8
2022	2.6

Notes: HIV= human immunodeficiency virus**.** Reference:

Figure A3. Annual number of HIV-associated deaths by health insurance coverage.

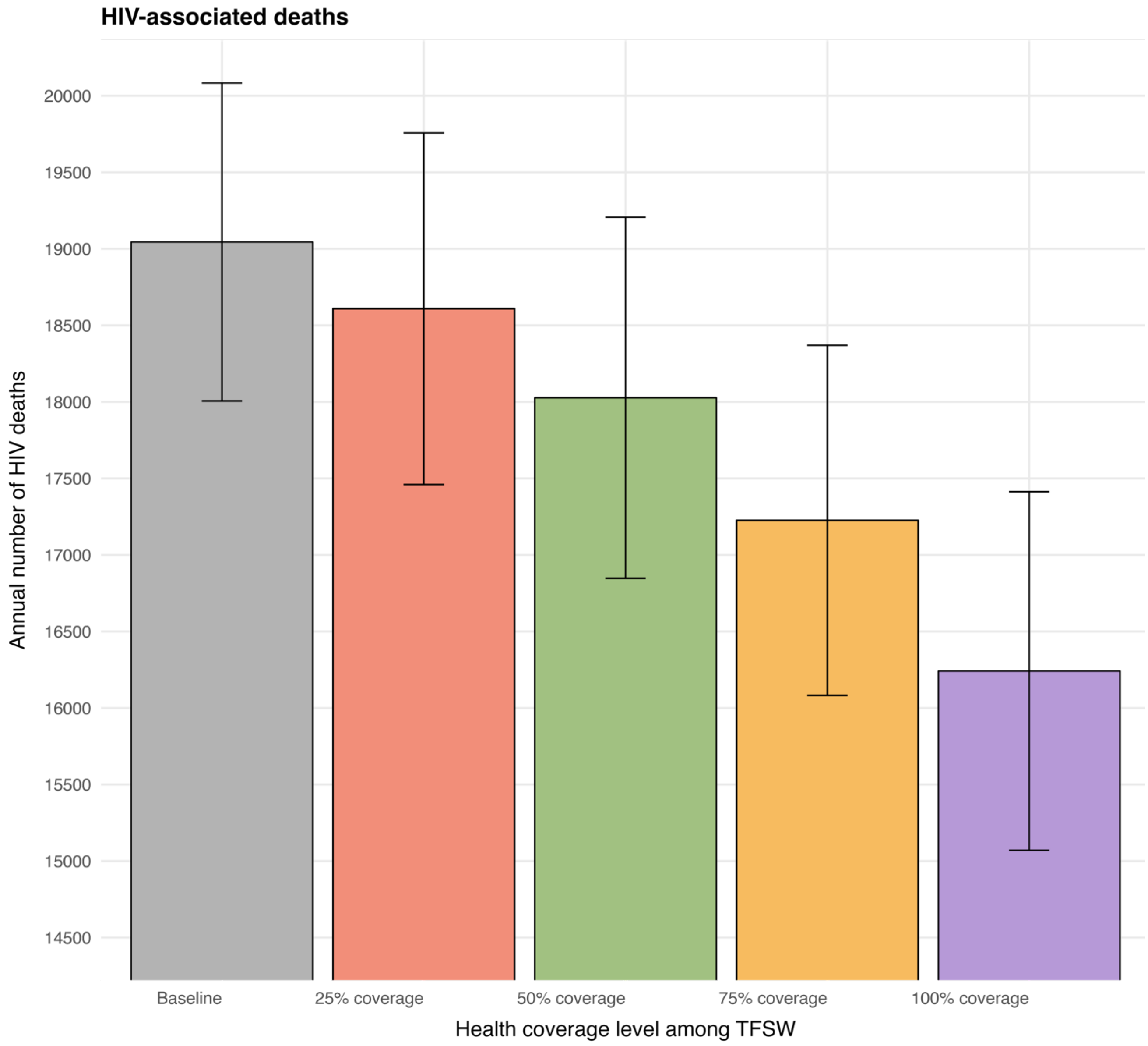

Notes: Confidence intervals were computed based on Cameroon's prevalence lower and upper bounds. Baseline means no coverage (0%).

Figure A4. Probability sensitivity analysis of incremental costs and DALYs and willingness-to-pay thresholds using different health insurance schemes among WGTS over 50 years.

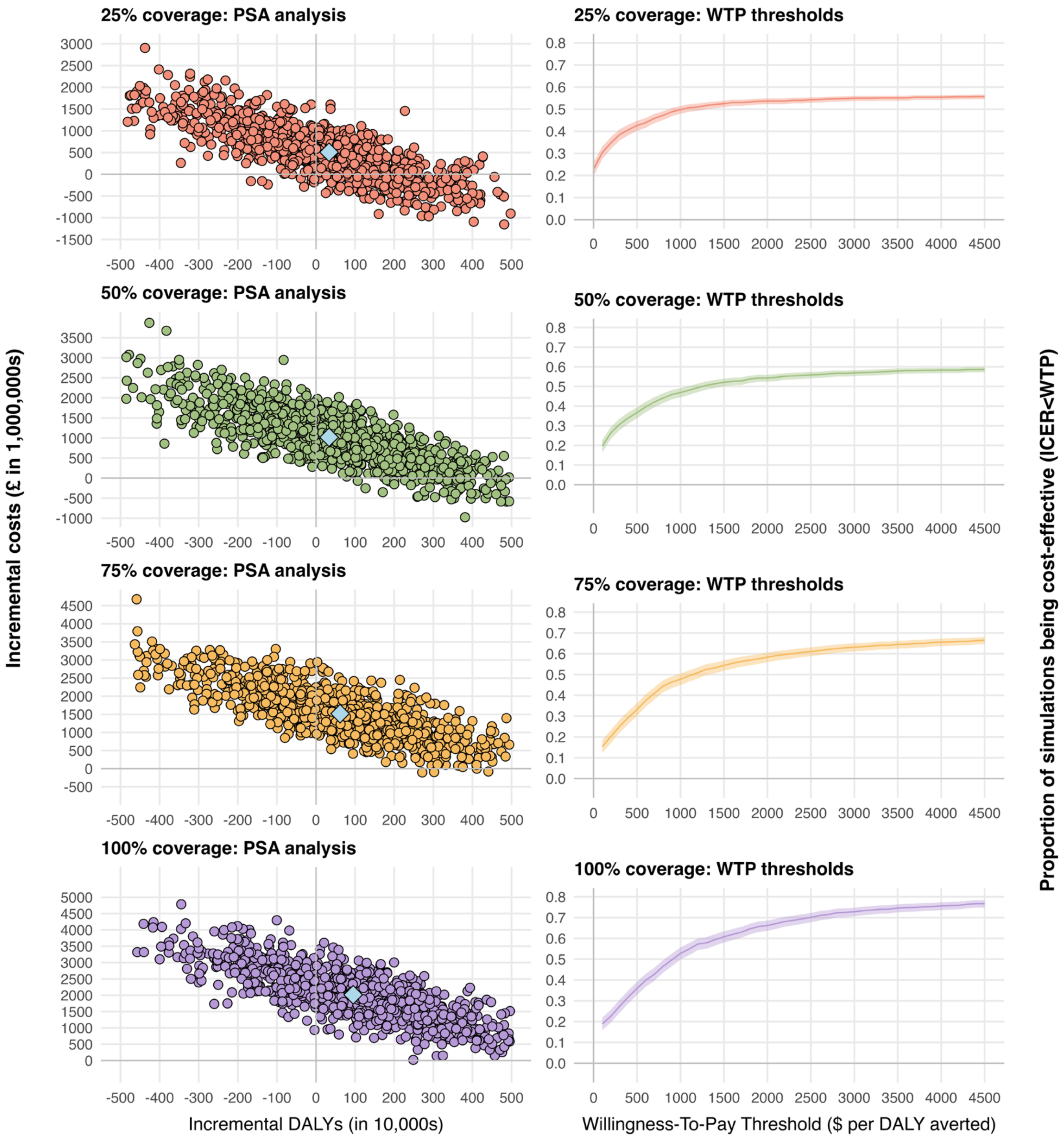

Notes: PSA: probability sensitivity analysis. WTP=Willingness-to-pay. sky-blue diamonds in PSA analysis shows the mean of incremental costs and infections averted. WTS= Women and girls engaging in transactional sex. DALYs= Disability-adjusted life years.

Figure A5. Probability sensitivity analysis of incremental costs and HIV infections and willingness-to-pay thresholds using different health insurance schemes among WGTSs over 50 years.

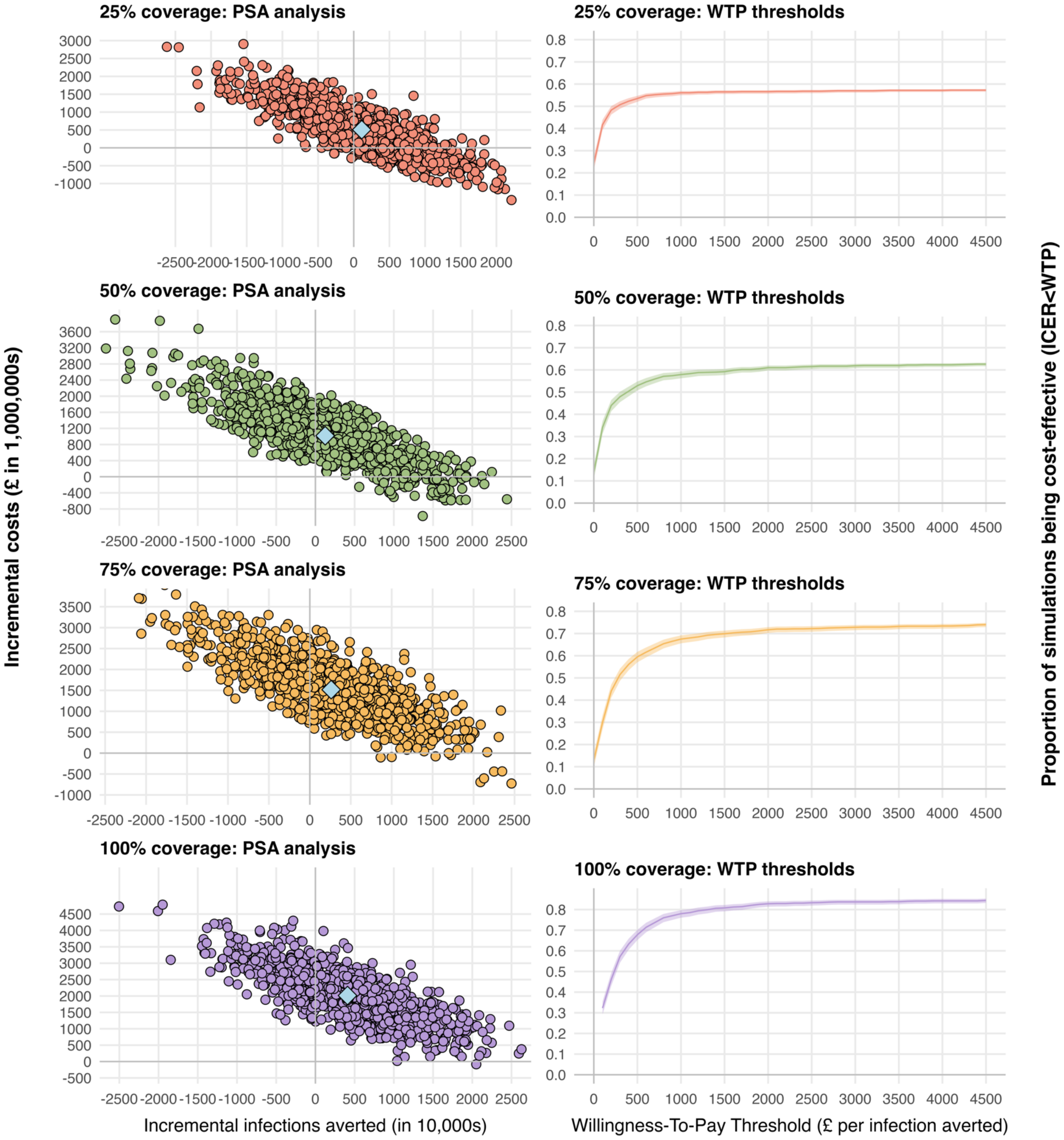

Notes: PSA: probability sensitivity analysis. WTP=Willingness-to-pay. sky-blue diamonds in PSA analysis shows the mean of incremental costs and infections averted. WGTS= Women and girls engaging in transactional sex.

Figure A6. (A) Global sensitivity analysis with most influential parameters over prevalence of HIV infected individuals and **(B)** Dual sensitivity analysis between health insurance coverage and efficiency over annual number of people HIV-infected.

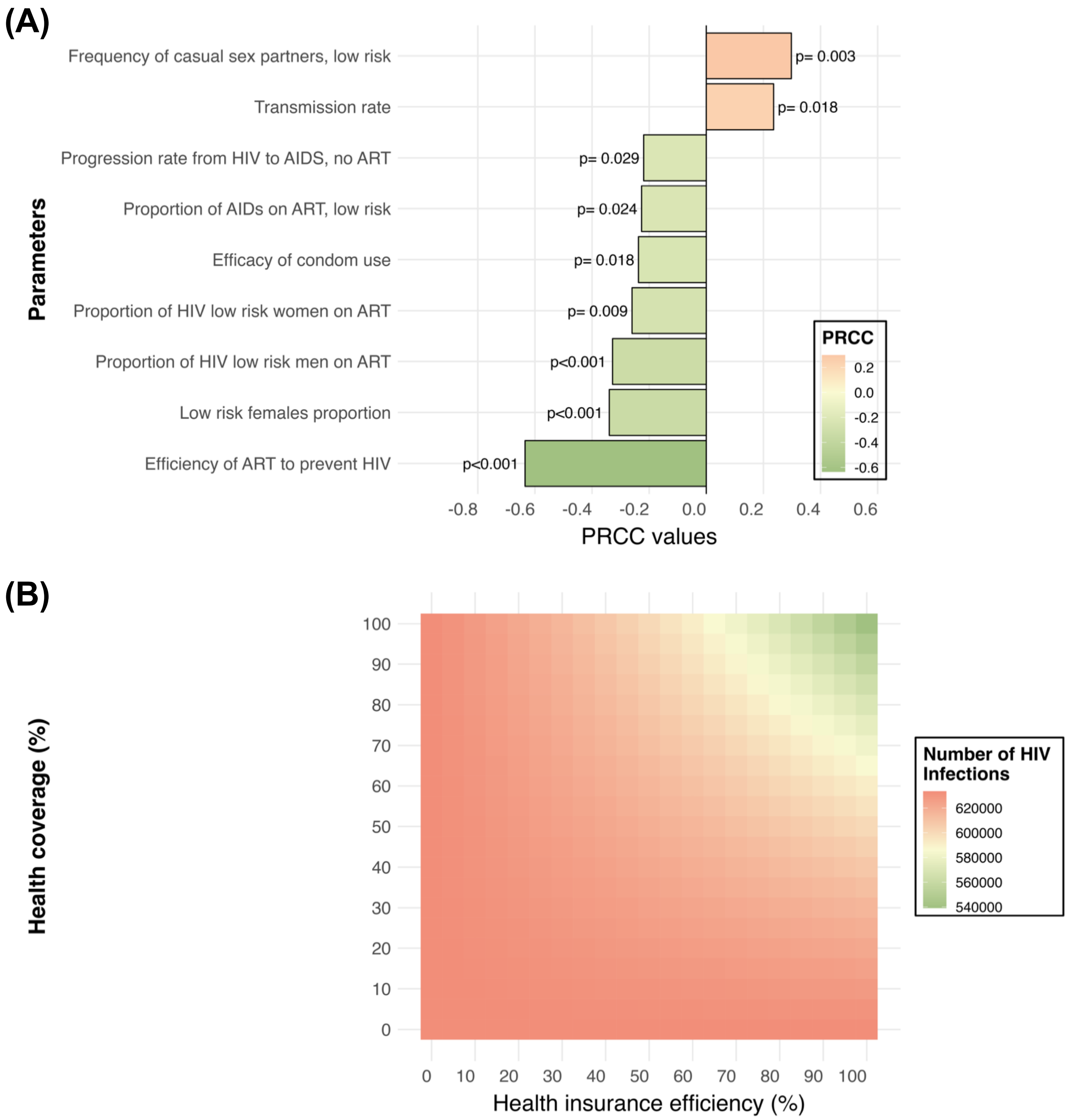

Notes: HIV= human immunodeficiency virus. WGTS= Women and girls engaging in transactional sex. MM= millions. Avg.= Average. ART= Antiretroviral therapy.

Figure A7. Sensitivity analyses for the annual number of people who died due to HIV by health insurance coverage and efficiency levels.

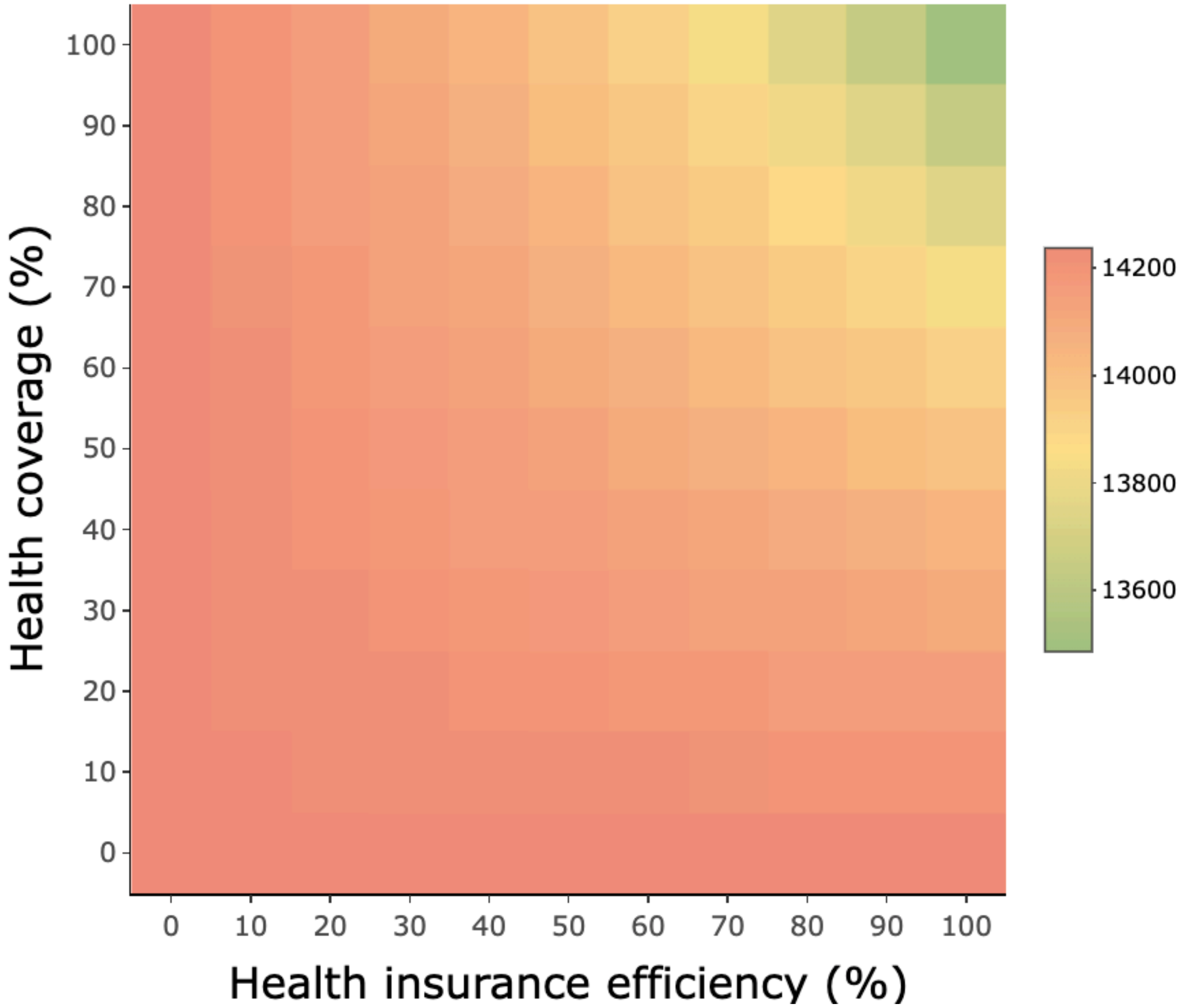

Notes: HIV= human immunodeficiency virus. WGTS= Women and girls engaging in transactional sex. MM= millions.

Table A5. PreP and Health insurance spending regarding hypothetical national expenditure considering our modelled population of WGTS and using different coverage levels (if administered separately)

**PrEP scheme only**					
Coverage (proportions)	0	0.25	0.5	0.75	1
Hypothetical government expenditure	0	£86,850,020	£173,700,041	£260,550,061	£347,400,081
As a % of the annual health expenditure in Cameroon	0	6.1%	12.2%	18.4%	24.5%
**Health insurance scheme only**					
Coverage (proportions)	0	0.25	0.5	0.75	1
Hypothetical government expenditure	0	£20,495,580	£40,991,160	£61,486,740	£81,982,320
As a % of the annual health expenditure in Cameroon	0	1.4%	2.9%	4.3%	5.8%

Considering an annual health expenditure in Cameroon per capita of £50[33] and the total population of 28,372,687 inhabitants,[34] we calculated national health expenditure as £1,418,634,350. In summary (Table A5), the hypothetical government expenditure for the PrEP scheme increases significantly with coverage, ranging from £86.85 million (6.1% of annual health expenditure) at 25% coverage to £347.4 million (24.5%) at full coverage. In contrast, the health insurance scheme is less costly, with expenditure ranging from £20.50 million (1.4%) at 25% coverage to £81.98 million (5.8%) at full coverage. These results suggest that the PrEP scheme requires substantially higher investment compared to the health insurance scheme at all levels of coverage.

Reflexivity statement -summary-

Kasim Allel led the mathematical modelling and conceptualisation of this study. KA, an informed "insider" with expertise in mathematical modelling, health economics, and HIV epidemiology, brought an understanding of the interplay between structural interventions and HIV prevention. This technical background allowed KA to critically assess the implications of the model's assumptions and limitations while maintaining an awareness of the broader policy context.

The mathematical model presented in this study is deeply rooted in the insights gained from a power study collaboratively conducted by University College London (UCL) and the Johns Hopkins Cameroon Program (JHCP). This collaboration brought together a multidisciplinary team of experts from both institutions, ensuring a diverse and robust analytical approach.

The authors of this paper include key contributors from both organisations who played leading roles in the conceptualisation, implementation, and analysis of the power study. From UCL, the team comprises Aurelia Lepine, Sandie Szawzloski, and Henry Cust, while the JHCP team includes Emile Nitcheu, Eric Defo Tamgno, Julienne Noo, Illiasou Mfochive, Serge Billong, and Ubald Tamoufe. Each of these individuals brought unique expertise and perspectives, enriching the collaborative effort and the subsequent development of the mathematical model.

This partnership reflects a commitment to integrating academic rigor with on-the-ground programmatic insights, ensuring the relevance and applicability of the findings to real-world contexts. By leveraging the strengths of both institutions, this study underscores the importance of interdisciplinary and cross-institutional collaboration in advancing research in global health.

The interdisciplinary nature of the research team ensured a comprehensive approach. HC and AL brought valuable insights from their experiences in global health and policy implementation, which informed the framing of the study's conclusions. The team collectively drew on data from the POWER study, ensuring the modelling was rooted in real-world contexts and informed by the nuanced realities of transactional sex in Cameroon.

While Kasim Allel's expertise facilitated the development of a robust model, it also necessitated deliberate efforts to remain objective and avoid over-interpretation of findings. Regular team discussions were held to critically evaluate the model's outputs and their applicability in diverse policy scenarios, ensuring the conclusions were grounded in evidence and relevant to the target populations.

The researchers acknowledge the inherent challenges in studying marginalized populations such as women engaging in transactional sex. Collaborative engagement with local stakeholders, including community-based organizations, was essential in aligning the study's focus with the lived realities of the target groups (essential part of the POWER RCT[3]). This process helped mitigate potential biases and ensured the research findings were both accurate and culturally sensitive.

Additionally, to reduce potential bias during the analysis, all team members contributed to a critical review of the assumptions, parameters, and interpretations of the model. This iterative process ensured that the limitations and applicability of the results were transparently communicated in the manuscript. By combining diverse perspectives and adhering to rigorous ethical and methodological standards, the team aimed to produce findings that are both scientifically robust and practically meaningful.

Reflexivity Statement questions following BMJ Global Health guidelines

How does this study address local research and policy priorities?

This study directly addresses critical gaps in HIV prevention among women and girls engaged in transactional sex (WGTS) in Cameroon, a population often excluded from key HIV interventions. By exploring the cost-effectiveness of health insurance as a structural intervention, the study aligns with national HIV policy goals and global efforts to enhance equitable healthcare access for marginalized populations.

How were local researchers involved in study design?

The mathematical model presented in this study is based on data collected as part of the POWER trial, a research initiative led by local researchers from the Johns Hopkins Cameroon Program (JHCP) in Yaoundé. The JHCP team was instrumental in the trial's design, implementation, and data collection processes. Their leadership ensured that the study was grounded in the local context, leveraging their in-depth understanding of the healthcare system, cultural dynamics, and population-specific factors. The involvement of JHCP researchers in the study design underscores the collaborative nature of this work and the pivotal role of local expertise in ensuring the relevance and applicability of the findings. By centring the trial in Yaoundé and involving local researchers at every stage, the study benefited from a contextually informed approach, which significantly enhanced the validity and utility of the data for the development of the mathematical model.

How has funding been used to support the local research team?

Funding for this project has played a critical role in supporting the local research team. Specifically, the UKRI Future Leaders Fellowship, awarded to the Principal Investigator (PI) Aurelia Lepine at University College London (UCL), has provided sustained financial support for the team of local researchers over a seven-year period. This funding has enabled the JHCP team to lead the trial, contribute to study design, manage data collection, and participate in capacity-building activities. It has also facilitated ongoing collaboration between UCL and JHCP, ensuring that the research reflects the local context while fostering long-term development within the local team. By investing in local researchers, the project has strengthened the capacity for independent research and innovation within Cameroon, aligning with the goals of equitable and sustainable global health research partnerships.

How are research staff who conducted data collection acknowledged?

Data collection for this study relied on the POWER randomized controlled trial. All researchers who contributed to data collection, including survey administration and stakeholder interviews are coauthors of the paper, all of which provided feedback accordingly.

Do all members of the research partnership have access to study data?

All collaborating researchers, including those based in Cameroon, had access to the anonymised study data to ensure transparency and inclusivity in the analysis and interpretation of findings. The POWER study was published previously as part of the project.[3]

How was data used to develop analytical skills within the partnership?

Local researchers collaborated in data analysis and interpretation, receiving hands-on training in mathematical modelling and sensitivity analysis. This approach enhanced their analytical capabilities and understanding of modelling outcomes, promoting skill transfer and research equity.

How have research partners collaborated in interpreting study data?

Interpretation of study data involved iterative discussions among the research team, combining local expertise and technical modelling knowledge. These discussions ensured a balanced perspective, addressing both the quantitative outputs and the social implications of the findings.

How were research partners supported to develop writing skills?

Junior researchers were mentored by senior authors during the drafting process, gaining experience in scientific writing. Local team members were provided with feedback and guidance to refine their contributions and develop their academic writing skills.

How will research products be shared to address local needs?

The study findings will be disseminated openly through publication in an open-access journal. In addition, we will collaborate with local stakeholders, including policymakers and community health organizations, to ensure the research informs targeted interventions for WGTS in Cameroon. Specifically, we will organise a national workshop in Cameroon to disseminate the results of this study to key national stakeholders, including policymakers, healthcare professionals, and researchers. This workshop will provide a platform to present findings, discuss their implications, and explore how they can inform policy and practice within the local context.

How is the leadership, contribution, and ownership of this work by LMIC researchers recognized within the authorship?

Researchers based in Cameroon are included as co-authors, reflecting their essential contributions to study design, data interpretation, and contextual analysis. Leadership roles within the team were distributed to ensure fair recognition of their expertise.

How have early-career researchers across the partnership been included within the authorship team?

Researchers participated in the manuscript development, so they were added as part of the author list.

How has gender balance been addressed within the authorship?

The authorship team comprises researchers of diverse genders, with an effort to include both male and female researchers from Cameroon and partner institutions.

How has the project contributed to the training of LMIC researchers?

The project has facilitated hands-on training in data collation and analysis for researchers in Cameroon.

How has the project contributed to improvements in local infrastructure?

While this project has not directly contributed to infrastructure development, it has enhanced local research capacity and provided actionable insights to inform healthcare policy improvements.

What safeguarding procedures were used to protect local study participants and researchers?

The study used secondary data and did not involve primary data collection. Ethical safeguards were adhered to during the analysis, and the anonymity of participants in the POWER study was maintained throughout the research process.

References

1. Stone J, Bothma R, Gomez GB, et al. Impact and cost-effectiveness of the national scale-up of HIV pre-exposure prophylaxis among female sex workers in South Africa: a modelling analysis. *Journal of the International AIDS Society* 2023; **26**(2): e26063.
2. Mukandavire C, Walker J, Schwartz S, et al. Estimating the contribution of key populations towards the spread of HIV in Dakar, Senegal. *Journal of the International AIDS Society* 2018; **21**: e25126.
3. Lépine A, Szawlowski S, Nitcheu E, et al. Protecting women from economic shocks to prevent HIV in Africa: Evidence from the POWER randomised controlled trial in Cameroon. *PLoS Medicine* 2024: 21.10: e1004355.
4. Bekolo CE, Kouanfack C, Ateudjieu J, et al. The declining trend in HIV prevalence from population-based surveys in Cameroon between 2004 and 2018: myth or reality in the universal test and treat era? *BMC Public Health* 2023; **23**(1): 479.
5. World Bank. Population ages 15-64 (% of total population) - Cameroon. 2024. https://data.worldbank.org/indicator/SP.POP.1564.TO.ZS?locations=CM.
6. world Bank. Population, total - Cameroon. 2024. https://data.worldbank.org/indicator/SP.POP.TOTL?locations=CM.
7. World Bank. Population, female (% of total population) - Cameroon. 2024. https://data.worldbank.org/indicator/SP.POP.TOTL.FE.ZS?locations=CM (accessed 8 February 2024).
8. Carael M, Slaymaker E, Lyerla R, Sarkar S. Clients of sex workers in different regions of the world: hard to count. *Sexually transmitted infections* 2006; **82**(suppl 3): iii26-iii33.
9. Billong SC, Nguefack-Tsague G, Fokam J, et al. Mapping and size estimates of female sex workers in Cameroon: Toward informed policy for design and implementation in the national HIV program. *PloS one* 2019; **14**(2): e0212315.
10. Silhol R, Baral S, Bowring AL, et al. Quantifying the evolving contribution of HIV interventions and key populations to the HIV epidemic in Yaoundé, Cameroon. *JAIDS Journal of Acquired Immune Deficiency Syndromes* 2021; **86**(4): 396-405.
11. Rucinski KB, Schwartz SR, Mishra S, et al. High HIV prevalence and low HIV-service engagement among young women who sell sex: a pooled analysis across 9 Sub-Saharan African countries. *JAIDS Journal of Acquired Immune Deficiency Syndromes* 2020; **85**(2): 148-55.
12. UNAIDS. Country factsheets: Cameroon. 2023. https://www.unaids.org/en/regionscountries/countries/cameroon (accessed 5th of September 2023).
13. Pinkerton SD, Abramson PR. Effectiveness of condoms in preventing HIV transmission. *Social science & medicine* 1997; **44**(9): 1303-12.
14. Cohen MS, Chen YQ, McCauley M, et al. Antiretroviral therapy for the prevention of HIV-1 transmission. *New England Journal of Medicine* 2016; **375**(9): 830-9.
15. Rutherford GW, Lifson AR, Hessol NA, et al. Course of HIV-I infection in a cohort of homosexual and bisexual men: an 11 year follow up study. *British Medical Journal* 1990; **301**(6762): 1183-8.
16. Okwundu CI, Uthman OA, Okoromah CA. Antiretroviral pre-exposure prophylaxis (PrEP) for preventing HIV in high-risk individuals. *Cochrane database of systematic reviews* 2012; (7).

17. Poka-Mayap V, Pefura-Yone EW, Kengne AP, Kuaban C. Mortality and its determinants among patients infected with HIV-1 on antiretroviral therapy in a referral centre in Yaounde, Cameroon: a retrospective cohort study. *BMJ open* 2013; **3**(7): e003210.
18. Luma HN, Mboringong F, Doualla M-S, et al. Mortality in hospitalised HIV/AIDS patients in a tertiary centre in sub-saharan africa: trends between 2007 and 2015, causes and associated factors. *The Open AIDS Journal* 2018; **12**(1).
19. Hendrickson C, Long LC, van Rensburg C, et al. The early-stage comprehensive costs of routine PrEP implementation and scale-up in Zambia. *PLOS Global Public Health* 2022; **2**(11): e0001246.
20. Tagar E, Sundaram M, Condliffe K, et al. Multi-country analysis of treatment costs for HIV/AIDS (MATCH): facility-level ART unit cost analysis in Ethiopia, Malawi, Rwanda, South Africa and Zambia. *PloS one* 2014; **9**(11): e108304.
21. Salomon JA, Haagsma JA, Davis A, et al. Disability weights for the Global Burden of Disease 2013 study. *The Lancet Global Health* 2015; **3**(11): e712-e23.
22. World Bank. Birth rate, crude (per 1,000 people) - Cameroon. 2024. https://data.worldbank.org/indicator/SP.DYN.CBRT.IN?locations=CM (accessed 8 February 2024).
23. Ngugi EN, Roth E, Mastin T, Nderitu M, Yasmin S. Female sex workers in Africa: epidemiology overview, data gaps, ways forward. *SAHARA-J: Journal of Social Aspects of HIV/AIDS* 2012; **9**(3): 148-53.
24. Mziray EN. Mapping and size estimation of female sex workers in Cameroon: to inform HIV program design and implementation-executive summary: The World Bank, 2016.
25. World Bank. Death rate, crude (per 1,000 people) - Cameroon. 2024. https://data.worldbank.org/indicator/SP.DYN.CDRT.IN?locations=CM (accessed 8 February 2024).
26. UNAIDS. HIV and AIDS - Basic facts General,. 2024. https://www.unaids.org/en/frequently-asked-questions-about-hiv-and-aids#:~:text=The%20majority%20of%20people%20infected,and%20not%20categorized%20as%20AIDS. (accessed 8 February 2024).
27. Trickey A, Sabin CA, Burkholder G, et al. Life expectancy after 2015 of adults with HIV on long-term antiretroviral therapy in Europe and North America: a collaborative analysis of cohort studies. *The Lancet HIV* 2023; **10**(5): e295-e307.
28. Rao A, Baral S, Phaswana-Mafuya N, et al. Pregnancy intentions and safer pregnancy knowledge among female sex workers in Port Elizabeth, South Africa. *Obstetrics & Gynecology* 2016; **128**(1): 15-21.
29. SAHMS-FSW. South African Health Monitoring Survey (SAHMS): an integrated biological and behavioural survey among female sex workers, South Africa 2013–2014. 2014.
30. Quaife M, Eakle R, Cabrera M, et al. Preferences for ARV-based HIV prevention methods among men and women, adolescent girls and female sex workers in Gauteng Province, South Africa: a protocol for a discrete choice experiment. *BMJ open* 2016; **6**(6).
31. Janes H, Corey L, Ramjee G, et al. Weighing the evidence of efficacy of oral PrEP for HIV prevention in women in Southern Africa. *AIDS Research and Human Retroviruses* 2018; **34**(8): 645-56.
32. Ajeh RA, Gregory HE, Thomas EO, et al. Determinants of retention in HIV antiretroviral treatment (ART) in the Cameroon International epidemiology Database to Evaluate AIDS (IeDEA) study clinics: the context of the HIV treat all strategy in Cameroon. *Pan African Medical Journal* 2021; **40**(1).

33. World Bank. Current health expenditure per capita (current US$) - Cameroon. 2023. https://datos.bancomundial.org/indicador/SH.XPD.CHEX.PC.CD?locations=CM (accessed 12 December 2024).
34. World Health Organization. Data : Cameroon. 2023. https://data.who.int/countries/120 (accessed 12 December 2024).
